# Optimal interphase delay in biphasic current pulses facilitates neural circuit activation induced by microstimulation in the mouse visual cortex

**DOI:** 10.3389/fnins.2025.1710221

**Published:** 2026-01-12

**Authors:** Santa Fukuda, Yuki Hayashida

**Affiliations:** Department of Information Engineering, Graduate School of Engineering, Mie University, Tsu, Mie, Japan

**Keywords:** biphasic current pulse, brain slice, interphase delay, intracortical microstimulation, mouse visual cortex, neural prostheses, voltage-sensitive dye imaging

## Abstract

Cathodic-first biphasic current pulses are commonly employed in intracortical microstimulation. Due to the intricate arrangement of axons, somata, and dendrites in the cerebral cortex, membrane polarizations induced by the cathodic and subsequent anodic phases of biphasic pulses may interact in a location-, magnitude-, and timing-dependent manner. Introducing an interphase delay between the two phases has been proposed to mitigate counteracting interactions between these membrane polarizations. Previous clinical studies on visual prostheses have demonstrated that such a delay lowers the stimulus threshold required for percept induction. However, despite this functional outcome, direct physiological observations of cathodic–anodic interactions in cortical circuit activation remain limited. Here, we employed voltage-sensitive dye imaging in mouse brain slices to visualize membrane excitation elicited by biphasic current pulses with varying interphase delays. The results demonstrated that an optimal interphase delay nonlinearly facilitated cortical circuit activation in response to both single and repetitive pulses. At 10 μA/phase and 200 μs/phase, the biphasic pulse elicited larger excitation with a 500–600-μs interphase delay than with shorter or longer delays or the cathodic monophasic pulse. At 20 μA/phase, the cathodic monophasic pulse and biphasic pulses with interphase delays > 800 μs elicited larger excitation than all other conditions. Pharmacological experiments suggested that trans-synaptic excitation contributes to this facilitative effect. These findings provide evidence that an optimally timed cathodic–anodic interaction enhances cortical circuit activation, beyond simply negating opposing phase effects. Such timing optimization of stimulus pulses may improve neural recruitment efficiency while minimizing charge delivery, offering insights for intracortical prosthetic design.

## 1 Introduction

Intracortical microstimulation (ICMS) is widely used to activate specific neural circuits within the brain. In clinical trials, ICMS has been delivered to sensory cortices to induce artificial sensations. (Bak et al., 1990; Schmidt et al., 1996; Flesher et al., 2016; Armenta Salas et al., 2018). More recently, the feasibility of intracortical visual prostheses has been demonstrated in both humans (Fernández et al., 2021) and non-human primates (Chen et al., 2020). However, clinical studies have also reported that the characteristics of artificial sensations and the required charge to elicit these sensations vary considerably depending on the stimulus parameters (Schmidt et al., 1996; Fernández et al., 2021; Flesher et al., 2016; Armenta Salas et al., 2018; Hughes et al., 2021; Fifer et al., 2022; Shelchkova et al., 2023; Hobbs et al., 2025; Greenspon et al., 2025; Valle et al., 2025). Optimizing these parameters is therefore a critical step toward improving the efficiency, safety, and power consumption of neural prostheses.

A cathodic-first biphasic current pulse is most commonly employed for ICMS (Schmidt et al., 1996; Flesher et al., 2016; Armenta Salas et al., 2018; Fernández et al., 2021). By maintaining charge balance at the electrode–tissue interface, biphasic pulses minimize electrode corrosion and reduce the risk of tissue damage (Lilly et al., 1955; Merrill et al., 2005). Importantly, cathodic and anodic currents exert distinct physiological effects, and these effects can interact depending on the stimulation parameters, as interpreted by previous computational studies (McIntyre and Grill, 2002; Grill, 2015). In addition, recent studies showed that trains of biphasic current pulses with different phase orders and/or with asymmetric amplitudes between the phases can induce distinct activities in specific neural populations (Bari et al., 2013; Stieger et al., 2020, 2022). Understanding such cathodic-anodic interactions at high temporal resolution is essential for the rational optimization of ICMS.

In general, cathodic currents induce membrane depolarization, whereas anodic currents produce hyperpolarization in the close vicinity of the electrode tip (Ranck, 1975; Merrill et al., 2005). In a cathodic-first biphasic pulse, depolarization elicited during the cathodic phase can be counteracted by subsequent anodic-phase hyperpolarization, thereby elevating the threshold for action potential generation compared with a cathodic monophasic pulse (Bromm and Frankenhaeuser, 1968; van den Honert and Mortimer, 1979; Miller et al., 2001; Rubinstein et al., 2002). Introducing an interphase delay between the two phases can mitigate this cancelation effect (van den Honert and Mortimer, 1979). With sufficiently long delays, the anodic phase is unable to cancel already-initiated action potentials (Weitz et al., 2014; Joshi et al., 2017; Ghaffari et al., 2020).

Even in relatively simple neural structures such as axon layers or nerve fibers, these interactions can still be complex. A cathodic phase can depolarize membrane segments close to the electrode tip while simultaneously hyperpolarizing more distant sites (Ranck, 1975; Rattay, 1989; Merrill et al., 2005). Such distal hyperpolarization may hinder action potential propagation (Tai et al., 2009; Van de Steene et al., 2020). Conversely, an anodic phase induces distal depolarization, which can counteract cathodic-induced hyperpolarization and thereby facilitate action potential propagation (Zheng et al., 2022). In addition, due to the impact of virtual cathodes during the anodic currents, there are circumstances under which anodic currents have lower thresholds to activate some fibers compared to cathodic currents (Anderson et al., 2019). These highlight that the depolarizations and hyperpolarizations induced by cathodic and anodic currents interact in a location-, magnitude-, and timing-dependent manner (Eickhoff and Jarvis, 2021; Cappaert et al., 2013).

In the cerebral cortex, where complex networks of axons, somata, dendrites, and diverse neuron types coexist, such interactions could involve nonlinear dynamics to an even greater extent. Several clinical and animal studies have employed interphase delays of tens of microseconds to reduce cathodic–anodic counteraction (human: Schmidt et al., 1996; Fernández et al., 2021; Flesher et al., 2016; Hughes et al., 2021; Fifer et al., 2022; Shelchkova et al., 2023; Hobbs et al., 2025; Greenspon et al., 2025; Valle et al., 2025, non-human primates: Davis et al., 2012; Chen et al., 2020; Meikle et al., 2023, rodents: Allison-Walker et al., 2021; Eles and Kozai, 2020; Urdaneta et al., 2021; Meikle et al., 2022; Hughes and Kozai, 2023; Wu et al., 2023; Dadarlat et al., 2024). Notably, a study in the rat motor cortex demonstrated that biphasic pulses with interphase delays elicited larger limb displacements than pulses without delay (Deprez et al., 2018), consistent with psychophysical observations in clinical trials of intracortical visual prostheses (Schmidt et al., 1996, see p. 512, the last paragraph in the left column). Nevertheless, direct physiological evidence regarding the interactions between the delayed anodic currents and preceding cathodic currents in the cortices remains limited.

The present study addresses this gap by experimentally characterizing the effects of interphase delays in biphasic current pulses on spatiotemporal neural excitation in the primary visual cortex by using voltage-sensitive dye (VSD) imaging in acute brain slices (Tanaka et al., 2019). Our experimental results demonstrated that the insertion of an optimal interphase delay resulted in nonlinear facilitation of neural circuit activation, in addition to negating the cathodic–anodic counteraction. This work provides physiological evidence for the facilitative effect of interphase delays in biphasic current pulses in cortical tissue and establishes a foundation for optimizing ICMS parameters in the development of intracortical visual prostheses.

## 2 Materials and methods

C57BL/6J mice (4–7 weeks old at the time of experiments, female, *n* = 24) were used. Mice were obtained at 3–4 weeks of age from CLEA Japan, Inc. (Tokyo, Japan), and housed individually or in groups of up to three with free access to food and water under controlled conditions (22 °C ± 1 °C, 55% ± 5% humidity, 12 h light/dark cycle; lights on at 07:00) in Experimental Animal Facilities of Mie University until the experiments. Mice were euthanized by isoflurane overdose (≥5% in ambient air) in an induction chamber until complete loss of reflexes and cessation of respiration, followed by decapitation as a secondary method. All animal care and experimental procedures were approved by the Animal Experiment Committee of Mie University (approval no. 2021-19-*Sai*1-*Hen*1), and conducted in conformity with the Guidelines for Proper Conduct of Animal Experiments by the Science Council of Japan.

Voltage-sensitive dye imaging was performed as described in our previous studies (Tanaka et al., 2019). Detailed methods for VSD imaging setup and data analyses are described in the Supplementary Methods. Briefly, coronal cerebral tissue slices (300 μm thick) containing the primary visual cortex (V1) were prepared from a mouse brain after decapitation, and stained with VSD (NK3630, 31.25 μg/ml). The stained tissue slice was mounted in a recording chamber perfused with oxygenated artificial cerebrospinal fluid (aCSF), and illuminated through cover glass at the bottom of the chamber and an optical bandpass filter (694 ± 10 nm) with using a halogen lamp (U-LH 100 IR; Olympus, Tokyo, Japan). Optical signal transmitted through layer II/III of V1 along the medio-lateral axis was acquired at 1,000 fps with 12 × 64 binned pixels (∼154 × 820 μm) using an electron-multiplying (EM) charge-coupled device (CCD) camera (iXon 3 DU-897; Andor Technology, Belfast, UK). In several experiments, to block excitatory synaptic transmission in the cortex, the superfusate was supplemented with D-2-amino-5-phosphonovaleric acid (D-AP5, 20 μM), an NMDA-type glutamate receptor antagonist, and 6,7-dinitroquinoxaline-2,3-dione (DNQX, 10 μM), a non-NMDA-type glutamate receptor antagonist.

For current stimulation, we used a glass microelectrode with an open tip of ∼6 μm in diameter. The electrode tip was inserted into layer II/III of V1 at a depth of 100–150 μm from the cut surface. An Ag/AgCl pellet was placed at a distant location from the tissue slice in the recording chamber as the return electrode. Cathodic-first biphasic pulses with interphase delays ranging between 0 and 1 ms were applied as stimuli. For comparison, monophasic current pulses were also applied. The phase duration was set to 0.2 ms otherwise specified. Pulses were delivered either as single pulses or as trains at a repetition rate of 100 Hz.

The current charge was set to 2 or 4 nC/phase; correspondingly, the current amplitude was 10 or 20 μA/phase with the phase duration of 0.2 ms. Pulse waveforms and amplitudes were monitored by connecting a 10-kΩ resistor in series between the return electrode and the current return node of the stimulator, and recording the voltage across this resistor with an oscilloscope (TDS2024B; Tektronix, Beaverton, USA). Triggers for the EM-CCD camera, stimulator, and oscilloscope were controlled by a pulse generator (Master-8; A.M.P.I., Jerusalem, Israel).

Image processing was performed with MATLAB (ver. R2023a; MathWorks, Natick, USA) as described previously (Tanaka et al., 2019). The relative change in optical intensity at each pixel of the captured image was quantified as Δ*T*(*t*_*n*_)*/T*_0_, where *T*_0_ denotes the average transmission intensity before stimulation and Δ*T*(*t*_*n*_) denotes the change in transmission intensity from *T*_0_ at discrete time *t*_*n*_. Increases and decreases in Δ*T*(*t*_*n*_)*/T*_0_ correspond to membrane depolarization and hyperpolarization, respectively. For each trial, VSD signals in response to identical stimulation were obtained 15–180 times at 10–17 s intervals, and the corresponding VSD signals were averaged. The VSD signals in response to no stimulation were also obtained and subtracted from those obtained with stimulation. Each image of the subtracted VSD signals was processed offline by spatial filtering (Gaussian, 5 × 5-pixel mask, s.d. ≒ 1.07 pixel) to improve the signal-to-noise ratio. To preserve temporal dynamics of VSD signals, no temporal filtering was applied.

To compare the responses induced by cathodic monophasic pulse and cathodic-first biphasic pulses varying interphase delays, statistical analyses were performed with Python (ver. 3.10.0; Python Software Foundation, Wilmington, USA) using data exported from MATLAB in CSV file format. Non-parametric comparisons of related groups were performed using the Friedman test. If a significant difference was detected (*p* < 0.05), Conover’s *post-hoc* test with a Holm–Bonferroni correction was subsequently performed to identify specific differences between groups. Error bars in figures are standard error of the mean unless otherwise stated.

## 3 Results

### 3.1 Excitation elicited by single biphasic current pulses with interphase delay

**FIGURE 1:**
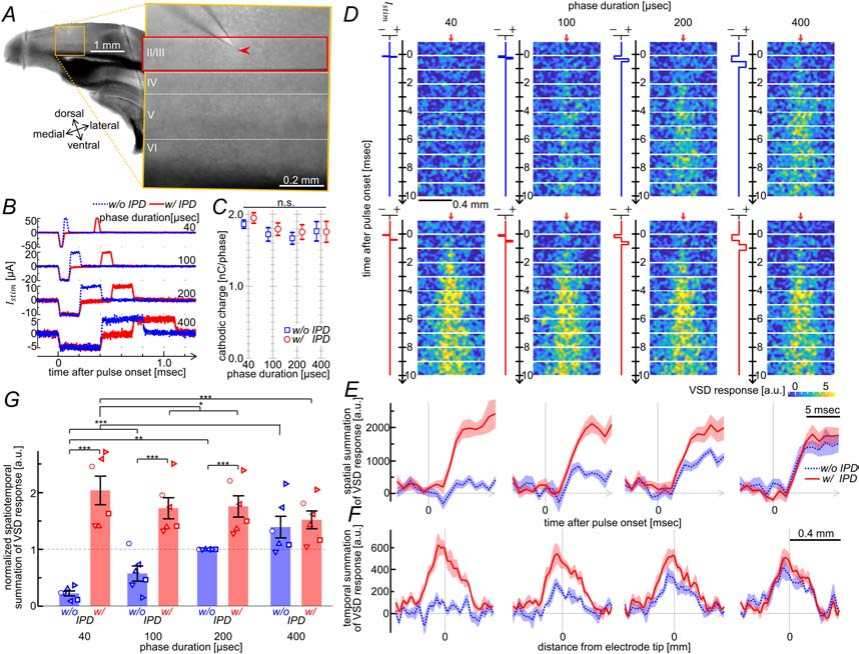
Effects of the insertion of an interphase delay in biphasic current pulses with different phase durations and an identical command stimulus change. **(A)** Infrared images of a cerebral slice at lower magnification (left) and of an area in V1 at high magnification (right). The dotted horizontal lines indicate approximate borders between cortical layers based on the somal shape, size, density, and distance from the cortical surface. Arrowhead indicates the stimulating electrode tip. **(B)** Overlaid traces of the stimulus current pulses with an interphase delay (red; “w/ IPD”) and without it (blue; “w/o IPD”) measured in one tissue slice. Note the differences in vertical axis scales. **(C)** Measured charge quantities in the cathodic phases of the current pulses shown in panel **(B)**. Marks indicate the means and error bars indicate the standard errors of the means (s.e.m.) across six slices. The Friedman test revealed no significant difference among eight biphasic pulses [*n* = 6, χ^2^(7) = 11.5, *p* = 0.12]. **(D)** Time-series images of VSD responses elicited by the current pulses shown in panel **(B)**, without (upper) and with (lower) an interphase delay. All images represent the average across the six slices. VSD response values (see Supplementary Methods) are color-coded according to the scale shown at bottom-right. An arrow at the top of each time-series image indicates the electrode tip position along the longitudinal axis. The blue or red waveform adjacent to each time axis represent the timing of the applied cathodic-anodic current phases. **(E,F)** Time courses of the spatially summated VSD responses **(E)** and spatial profiles of temporally summated VSD responses **(F)**, with (red) and without (blue) an interphase delay. Solid and dotted lines represent the means, and shadings represent the s.e.m. across the six slices. **(G)** Comparison of the spatiotemporal summation of VSD responses across stimulus conditions. Statistical significance was assessed using the Friedman test followed by Conover’s *post-hoc* test with Holm correction. Asterisks indicate significance (*p < 0.05, **p < 0.01, ***p < 0.001). Error bars represent s.e.m. across the six slices. For clarity, statistical significance is shown only for condition pairs sharing either the same phase duration or the same interphase delay. The complete pairwise matrix is provided in Supplementary Figure 1.

First, we verified that insertion of an interphase delay in biphasic current pulses can enhance the induction of neural excitation in the visual cortex slices by means of VSD imaging. Figure 1A shows an infrared image of the cerebral tissue slice (left) and its magnified view of an area including V1 (right), where the red box indicates the area of VSD imaging. As shown in the figure, the tip of a stimulating electrode was inserted into the layer II/III (arrowhead in the figure). Through the electrode, biphasic current pulses with the same command value of stimulus charge (2 nC/phase) but with four different phase durations (40, 100, 200, and 400 μs) were delivered to the tissue as stimuli (Figure 1B). In these pulses, the interphase delay was set to either 0 (blue traces in panel B) or 300 μs (red traces). Figure 1B shows the recorded traces of the current pulses injected into one tissue slice through the electrodes. Based on these recordings, the electrical charge quantities delivered by the cathodic phases of current pulses were measured, as shown in Figure 1C. The Friedman test revealed no significant differences in the charge of the cathodic phase among the eight biphasic pulses (*n* = 6, χ^2^(7) = 11.5, *p* = 0.12). This allowed us to compare the effects of current pulses on neural excitations between the conditions with and without the interphase delay. Figure 1D shows the time-series images of the VSD signal [i.e., Δ*T*(*t*_*n*_)/*T*_0_] in response to the stimuli, averaged across the six tissue slices. When no interphase delay was inserted in the current pulses (upper row in D), the stimuli elicited membrane excitations with amplitudes and spatial extents that depended on the phase durations of current pulses. The excitations were only faintly observed, particularly at the shortest phase duration of 40 μs. In contrast, when an interphase delay was inserted (lower row in D), the stimuli evoked marked membrane excitations even at the 40-μs phase duration. Also, the excitations elicited with longer phase durations were larger in amplitude and spatial extent than when no interphase delay was inserted (compare lower to upper rows in D). Figure 1E illustrates the time courses of the spatially summated VSD signals, calculated across all pixels in each image frame. The signal amplitude at time points from 5 to 10 ms after the pulse onset was 1.2 to 7.3 times larger with the interphase delay (red solid lines) than without it (blue dotted lines). Figure 1F illustrates the longitudinal-axis spatial profiles of temporally summated VSD signals, computed over a 10-ms duration following the pulse onset and averaged across all pixels along the transverse axis. The peak signal magnitude near the stimulation site was 1.2–4.3 times greater with the interphase delay (red solid lines) than without it (blue dotted lines). As shown in Figure 1G, multiple comparisons for the spatiotemporal summation of VSD signals over all pixels and for a 10-ms duration following the pulse onset supported that the insertion of a 300-μs interphase delay elicited significantly larger excitations than when no interphase delay was inserted at 40-, 100-, and 200-μs phase duration (*n* = 6, *p* < 7.9 × 10^–7^). For the 400-μs phase duration, the difference was not significant (*n* = 6, *p* = 0.18; see Supplementary Figure 1 for *p*-values for all pairwise comparisons). From a simplified electromagnetic perspective, the extracellular potential gradient induced by a cathodic-phase (or anodic-phase) current is reversed by the subsequent injection of an opposite-phase current. Because such changes in the potential gradient occur with specific time constants inherent to the tissue, the changes in membrane potential induced by the initial cathodic phase of a biphasic pulse may persist longer when the interphase delay is extended, before being countered by the following anodic phase. Therefore, within a certain temporal window following the cathodic phase, the membrane voltage may undergo greater depolarization, leading to action potential firing, as the interphase delay increases (van den Honert and Mortimer, 1979). If this hypothesis holds in our experimental preparation, a cathodic-phase current pulse—lacking a subsequent anodic phase—would be expected to produce the largest membrane depolarization. Based on this possibility, we compared the neural responses to monophasic current pulses and biphasic pulses with varying interphase delays in the following experiments.

### 3.2 Facilitation of excitation by an optimal interphase delay

For a systematic comparison, we used biphasic current pulses with interphase delays ranging from 0 to 1,000 μs in 200-μs increments, as well as monophasic current pulses. The phase duration was fixed at 200 μs, which has been widely used in previous studies (Brindley and Lewin, 1968; Stoney et al., 1968; Asanuma et al., 1976; Normann et al., 2002). The charge quantities measured for the cathodic (or anodic) phase of these current pulses varied by less than 9 %, ranging from 1.77 to 2.07 nC (see Section 10 in Supplementary Methods for details).

**FIGURE 2:**
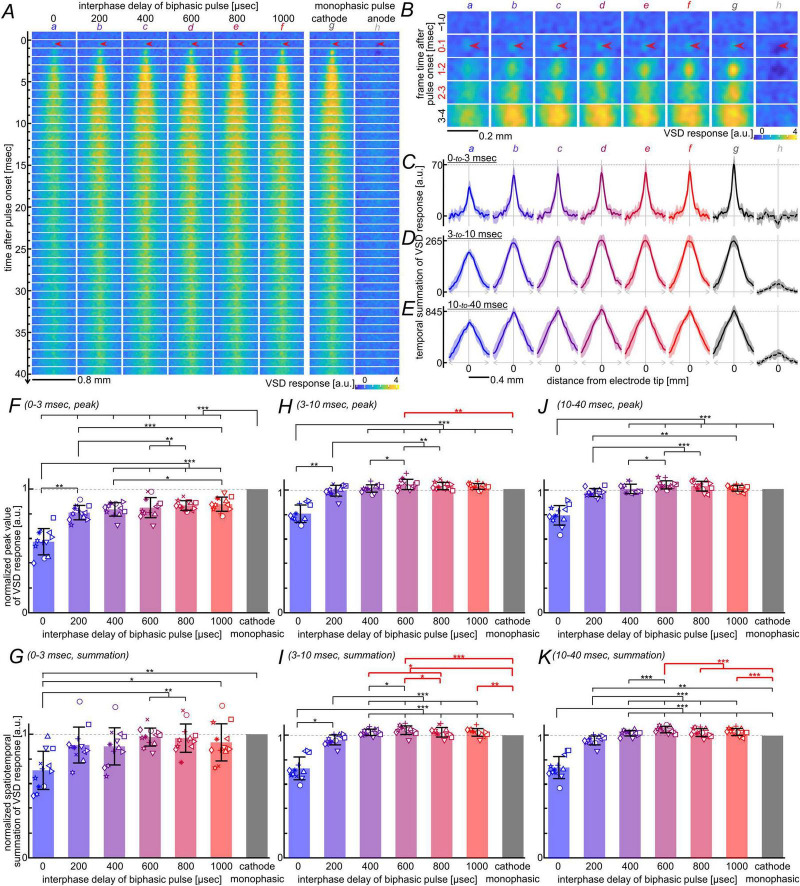
Comparison of neural excitation elicited by biphasic current pulses with varying interphase delays and monophasic current pulses. **(A)** Time-series images of VSD responses elicited by the current-pulse stimuli. The phase duration and the amplitude per phase were 200 μs and 10 μA, respectively. All images represent averages across 12 slices.VSD response values are color-coded according to the scale shown at the bottom. Arrowheads indicate the electrode tip position, spatially aligned across slices (see Supplementary Methods). The interphase delay was varied from 0 to 1,000 μs in 200-μs steps (a–f). Cathodic and anodic monophasic pulses are labeled g and h, respectively. **(B)** Expanded spatial views of VSD images extracted from panel **(A)**. Note the different color scale, as indicated at the bottom. Labels (a–h) correspond to those in panel **(A)**. **(C–E)** Spatial profiles of temporally summated VSD responses. Summation windows were 0–3 ms **(C)**, 3–10 ms **(D)**, and 10–40 ms **(E)** after pulse onset. Solid and dashed lines represent the means, and shadings represent the standard deviation (s.d.), across the twelve slices. **(F–K)** Comparison of peak amplitude **(F,H,J)** and temporal summation **(G,I,K)** of the response spatial profiles shown in panels **(C–E)**. Results shown in panels **(F–K)** were derived from the data shown in panels **(C–E)**, respectively. Statistical significance was evaluated using the Friedman test followed by Conover’s *post-hoc* test with Holm correction. Asterisks indicate significance (*p < 0.05, **p < 0.01, ***p < 0.001). Error bars represent s.d. across the twelve slices.

The time-series images of VSD signals in response to these current pulses are shown in Figure 2A. The data represent averages derived from twelve tissue slices, each examined with all eight pulse conditions. Consistent with the results shown in Figure 1, membrane depolarizations elicited by biphasic current pulses with interphase delays (b–f) were larger in amplitude than that elicited without a delay (a). To highlight the responses directly elicited by the current pulses, image frames from −1 to +4 ms after the pulse onset in Figure 2A were displayed at an expanded spatial scale in Figure 2B. The figure demonstrates that the biphasic current pulses with relatively long interphase delays (d–f) elicited larger depolarizations compared to the pulse without a delay (a). Moreover, the cathodic current pulse (g) elicited the largest depolarization among all stimuli (a–f). Also in this time range, the anodic current pulse (h) appeared to induce net membrane hyperpolarization, although the signal-to-noise ratio was relatively low. Similar results were obtained when synaptic transmission was blocked with glutamate receptor antagonists, D-AP5 and DNQX, in six out of the 12 tissue slices (Supplementary Figure 2B), suggesting that the facilitation of initial responses reflects enhanced action potential initiation with no anodic-phase current or prolonged interphase delays.

The spatial profiles of these initial responses were derived by temporally summating the VSD signals over 0–3 ms (i.e., three frames) after the pulse onset and averaging across all 11 pixels along the transverse axis, as shown in Figure 2C (see Supplementary Figure 3A for data from individual slices). Conover’s *post-hoc* test on the peak amplitudes of these profiles (Figure 2F) supported that the biphasic pulse without an interphase delay elicited the smallest peak response (*n* = 12, *p* < 0.0025), whereas the cathodic pulse elicited the largest peak response (*n* = 12, *p* < 4.8 × 10^–4^). In addition, to compare the overall magnitude of response, Conover’s *post-hoc* test was applied to the spatial summation of these profiles (Figure 2G). The analysis confirmed that the biphasic pulse without an interphase delay induced significantly smaller response magnitude than those with delays of 600–1,000 μs or the cathodic pulse (*n* = 12, *p* < 0.029; see Supplementary Figures 3B, C for details). In the presence of glutamate receptor antagonists, although the signal-to-noise ratio was much lower under synaptic blockade than under control conditions, statistical analyses of the data confirmed part of the results observed in the control condition; i.e., the significantly smaller response with no interphase delay than those with a delay of 1,000 μs, and with the cathodic pulse (Supplementary Figures 2C, F, G).

The above observations regarding the initial responses to biphasic and monophasic pulses are consistent with the electromagnetic perspective discussed in the previous subsection and with the conventional interpretation of the effect of inserting an interphase delay on action potential initiation (van den Honert and Mortimer, 1979).

After the initial responses to the current pulses, depolarizing responses progressively increased in both amplitude and spatial extent over time, as shown in the image frames from 3 to 10 ms after the pulse onset in Figure 2A. In this time window, the response to biphasic pulses with interphase delays of 600–800 μs (d, e) appeared similar to, or slightly larger in amplitude than, that elicited by the cathodic pulse (g), despite the smaller peak amplitudes of the initial responses. This tendency in response remained similar in the following time (i.e., 10–40 ms after the pulse onset in Figure 2A) while the depolarization gradually decayed over time. Additionally, the response to the anodic pulse (h in Figure 2A) turned from a hyperpolarizing to a small, spatially spread depolarizing response at 3–4 ms after the pulse onset, and this depolarization continued thereafter, possibly resulting from rebound and/or distal depolarizations (Merrill et al., 2005; Sun and Wu, 2008). The depolarizing response to a biphasic or monophasic pulse, occurring later than 3 ms after the pulse onset, was markedly suppressed by glutamate receptor antagonists (Supplementary Figure 2A), demonstrating post-synaptic/trans-synaptic excitations in these time ranges. The spatial profiles of these later responses, obtained by temporal summation of the VSD signals over 3–10 or 10–40 ms after the pulse onset and averaged across all 11 pixels along the transverse axis, are shown in Figures 2D, E (see Supplementary Figures 4A, 5A for data from individual slices). Conover’s *post-hoc* test applied to these profiles supported that the biphasic pulse without an interphase delay produced the smallest peak amplitude (Figures 2H, J) among all stimuli (*n* = 12; 3–10 ms: *p* < 0.0068; 10–40 ms: *p* < 1.0 × 10^–5^; see Supplementary Figures 4B, 5B for details), and the smallest magnitude of overall response (Figures 2I, K) among all stimuli except the biphasic pulse with a 200-μs delay (*n* = 12; 3-10 ms: *p* < 0.035; 10–40 ms: *p* < 4.1 × 10^–7^; see Supplementary Figures 4C, 5C for details). Intriguingly, the multiple comparisons showed that, in the 3–10-ms time range, the biphasic pulse with a 600-μs interphase delay elicited the peak amplitude that was larger than that elicited by the cathodic pulse (*n* = 12, *p* < 0.01; Figure 2H), and produced the largest magnitude of the overall response among all stimuli except the biphasic pulse with a 1,000-μs delay (*n* = 12, *p* < 0.025; Figure 2I). Also, in the 10–40-ms time range, the overall response magnitude was largest with the biphasic pulse with a 600-μs delay among all stimuli except the biphasic pulse with a 1,000-μs delay (*n* = 12, *p* < 1.8 × 10^–4^; Figure 2K). Such a non-linear facilitation of the later responses was markedly reduced by synaptic blockade (Supplementary Figures 2D, E), and statistical analyses of those data provided no significant difference between the responses with a 600-μs delay and the cathodic pulse (Supplementary Figures 2H–K). These observations suggested that a biphasic pulse with an interphase delay of 600 μs is more effective in evoking the postsynaptic/trans-synaptic membrane depolarization, compared to a monophasic cathodic pulse as well as biphasic pulses with shorter interphase delays.

As described above, the effects of inserting an interphase delay into a single biphasic pulse on neural excitation appeared to involve at least two contributing factors: the fostering of action potential initiation (Figures 2B, C, F, G and Supplementary Figure 2) and the delayed facilitation of membrane depolarization (Figures 2D, E, H–K and Supplementary Figures 4, 5). Since the latter occurred with slow dynamics spanning several to 10’s of milliseconds, its effect is expected to accumulate during repetitive pulse stimulation. Based on this possibility, we conducted the following experiments to compare neural responses to pulse-train stimuli using biphasic pulses with varying interphase delays.

### 3.3 Cumulative facilitation of excitation during pulse-train stimulation with optimal interphase delays

**FIGURE 3:**
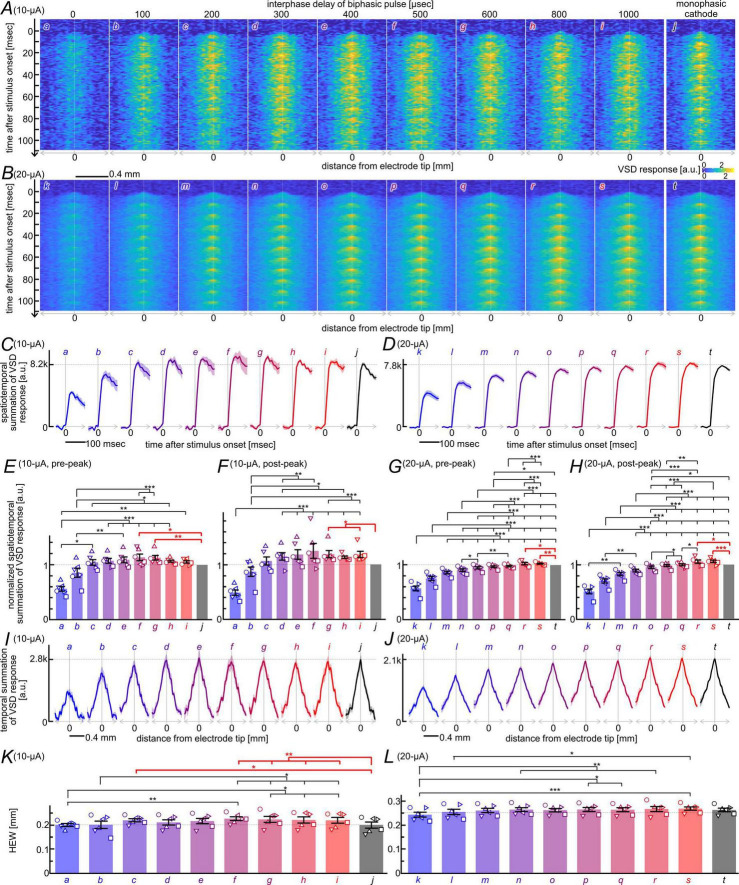
Comparison of neural excitation elicited by pulse-train stimulation. **(A,B)** Space-time plots of VSD responses elicited by trains of biphasic current pulses with varying interphase delays (a–i, k–s), and by trains of cathodic monophasic pulses (j,t). The phase duration was 200 μs, and the current amplitude was 10 μA/phase **(A)** or 20 μA/phase **(B)**. All images represent averages across six slices. VSD response values are color-coded according to the scale shown on the right. Interphase delay ranged from 0 to 1,000 μs, as indicated at the top, and are labeled (a–i) in panel **(A)** and (k–s) in panel **(B)**. **(C,D)** Time courses of the response magnitude. Labels (a–j) in panel **(C)** correspond to those in panel **(A)**, and labels (k–t) in panel **(D)** correspond to those in panel **(B)**. Solid lines and shadings represent the means and the s.e.m., respectively, across the six slices. **(E–H)** Comparison of the temporal summation of response magnitudes during the rising phase [“pre-peak”; **(E,G)**] and the subsequent phase [“post-peak”; **(F,H)**]. The current amplitude was 10 μA/phase **(E,F)** or 20 μA/phase **(G,H)**. **(I,J)** Spatial profiles of temporally summated VSD responses over 0–110 ms after pulse-train onset. The current amplitude was 10 μA/phase (I) or 20 μA/phase (J). Solid lines and shadings represent the means and the s.e.m., respectively, across the six slices. **(K,L)** Comparison of the half-energy widths of the spatial profiles shown in panels **(I,J)**. Statistical significance was evaluated using the Friedman test followed by Conover’s *post-hoc* test with Holm correction. Asterisks indicate significance (*p < 0.05, **p < 0.01, ***p < 0.001).

To examine cumulative effects of interphase delays, each stimulus condition consisted of a 100-Hz train of 11 identical pulses, using biphasic currents with interphase delays of 0–1,000 μs (a–i, k–s) or monophasic cathodic currents (j, t). In addition, the current amplitude was either 10 or 20 μA/phase. The data represent averages obtained from six tissue slices, each subjected to all 20 stimulation conditions. Figures 3A, B shows the time series of longitudinal-axis spatial profiles, calculated by averaging the VSD signals across 11 pixels along the transverse axis for each image frame (referred to as “space-time plot”; see Supplementary Methods for details). In response to each current pulse, delivered at 10-ms intervals, membrane depolarization was initiated near the stimulating electrode tip and subsequently propagated toward the periphery. The response amplitudes appeared greater in magnitude when an interphase delay was inserted (b–i in A and l–s in B) than when no interphase delay was applied (a in A, and k in B), consistent with the responses observed with single-pulse stimulation (Figure 2).

To quantitatively assess the effects of interphase delay, we first characterized the time course of response magnitude for each stimulation condition, as shown in Figures 3C, D (see Supplementary Figures 6A, B for data from individual slices). The response magnitude was calculated by spatiotemporally summating the longitudinal-axis profiles over a 10-ms window following each pulse onset. Across all interphase-delay conditions, the response magnitudes reached their peaks at 40–50 ms after the pulse-train onset for the 10-μA/phase condition (Figure 3C), and at 50–60 ms for the 20-μA/phase condition (Figure 3D). The peak response magnitude was approximately twice as large when interphase delays of 300–800 μs (10-μA/phase; d–h in C), or 800–1,000 μs (20-μA/phase; r–s in D) were inserted, compared with no interphase delay (a in C, and k in D).

Each time course was divided into a rising phase (0–40/50 ms, from the pulse-train onset to the peak of the response) and a subsequent phase (40/50–110 ms, from the peak to the end of the analysis window), and statistical analyses were performed on the temporal summation of response magnitudes within each phase (Figures 3E–H). Across all interphase-delay conditions, biphasic pulses without an interphase delay produced significantly smaller excitation magnitudes than those produced by pulses with delays of 300–1,000 μs in both temporal phases (*n* = 6; rising phase: *p* < 0.0036; subsequent phase: *p* < 5.3 × 10^–4^; see Supplementary Figures 6C–F for details). Moreover, in line with the results from the single-pulse experiment, at 10 μA/phase (Figures 3E, F), the biphasic pulse with a 600-μs interphase delay produced significantly larger excitation magnitudes than the cathodic pulse in both temporal phases (*n* = 6; rising phase: *p* = 0.0056; subsequent phase: *p* = 0.019; see Supplementary Figures 6C, D for details). Also, at 20 μA/phase (Figures 3G, H), biphasic pulses with interphase delays of 800–1,000 μs produced significantly larger excitation magnitudes than the cathodic pulse and biphasic pulses with all other interphase delays tested, again in both temporal phases (*n* = 6; rising phase: *p* < 0.014; subsequent phase: *p* < 0.011; see Supplementary Figures 6E, F for details). Additionally, at 10 μA/phase (Figures 3E, F), the excitation magnitude was significant larger for biphasic pulses with a 500-μs delay during the rising phase, and with a 1,000-μs delay during the subsequent phase, when compared with those with the cathodic pulses. Overall, these temporal analyses demonstrate that appropriately timed interphase delays substantially enhance the cumulative response magnitude during pulse-train stimulation.

We next characterized the overall spatial profile of the integrated response magnitude for each stimulation condition, as shown in Figures 3I, J (see Supplementary Figures 7A, B for data from individual slices). Each profile was obtained by integrating the longitudinal-axis spatial profile (Figures 3A, B) over the entire 110-ms period from the pulse-train onset, and statistical analyses were performed on the half-energy widths (HEW) of these overall profiles (Figures 3K, L). At 10 μA/phase (Figure 3K), biphasic pulses with no interphase yielded significantly narrower excitation widths than those with delays of 500–1,000 μs. Moreover, biphasic pulses with 500–1,000-μs delays produced significantly larger excitation widths than the cathodic pulse (*n* = 6, *p* < 0.046; see Supplementary Figure 7C for details). At 20 μA/phase (Figure 3L), significant differences in excitation width were observed between the biphasic pulse with no interphase delay and those with 300- or 500–1,000-μs delays (*n* = 6, *p* < 0.02), whereas no significant difference was detected between the cathodic pulse and any biphasic pulse condition (see Supplementary Figure 7D for details). These spatial analyses further indicate that interphase delay shapes not only the magnitude but also the extent of neural recruitment during pulse-train stimulation.

These results suggest that optimal interphase delays not only enhance the initial response to each pulse but also drives a cumulative increase in response magnitude over the first several pulses of the train. The shift in the optimal delay with stimulus amplitude further implies that this facilitative effect depends on the ongoing state of circuit activity. Together with the results from the single-pulse experiments (Figure 2 and Supplementary Figure 2), the cumulative facilitation is consistent with the involvement of trans-synaptic depolarization. To test this possibility, we analyzed responses recorded under synaptic blockade, as described in the following subsection.

### 3.4 Trans-synaptic contributions to the cumulative facilitation of excitation during pulse-train stimulation

**FIGURE 4:**
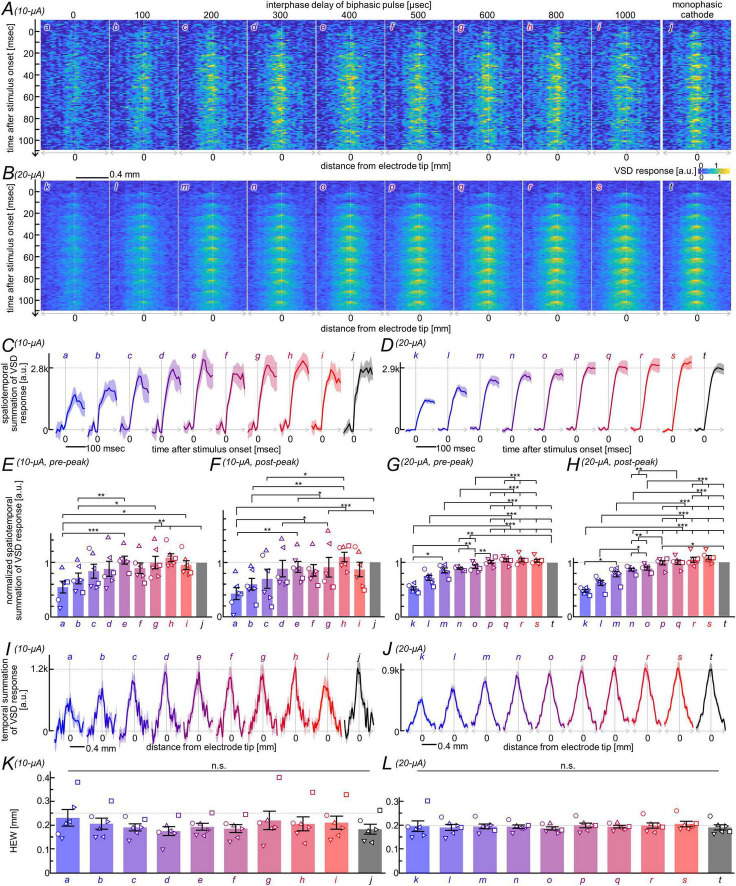
Comparison of neural excitation elicited by pulse-train stimulation under synaptic blockade. All data were obtained from the same slices examined in the experiment shown in Figure 3 while perfused with aCSF supplemented with glutamate receptor antagonists, D-AP5 and DNQX. The overall figure format and analysis procedures are identical to those used in Figure 3. **(A,B)** Space-time plots of VSD responses elicited by the pulse trains **(C,D)** Time courses of the response magnitude. **(E–H)** Comparison of the temporal summation of response magnitudes during the rising phase **(E,G)** and the subsequent phase **(F,H)**. **(I,J)** Spatial profiles of temporally summated VSD responses over 0–110 ms after pulse-train onset. **(K,L)** Comparison of the half-energy widths of the spatial profiles shown in panels **(I,J)**.

Here, we asked whether the nonlinear facilitation observed in the control condition persists under synaptic blockade with D-AP5 and DNQX in the same slices examined in Figure 3. Figures 4A, B shows the space–time plots of the responses to pulse-trains with an amplitude of 10 μA/phase (A) or 20 μA/phase (B). Because excitatory postsynaptic potentials and subsequent membrane depolarizations were suppressed by the antagonists, the VSD signal amplitudes were markedly reduced, resulting in a lower signal-to-noise ratio. Nevertheless, transient depolarizations elicited by each pulse in the train were still discernible in most conditions, consistent with the observations in the control condition. These depolarizing responses tended to increase in amplitude over the first several pulses of the train, although their spatial spread remained relatively restricted throughout the stimulation.

Similar to the analysis performed for the control condition, the time course of response magnitude was derived from the space–time plot for each stimulation condition (Figures 4C, D; see Supplementary Figures 8A, B for data from individual slices), and statistical analyses were performed on the temporal summation of response magnitudes during the rising phase (0–40/50 ms after train onset) and the subsequent phase (40/50–110 ms). In contrast to the control condition, Conover’s *post-hoc* test detected no significant difference between the cathodic pulse and biphasic pulses with interphase delays of 500–600 μs at the 10-μA/phase amplitude in either temporal phase (*n* = 6, *p* ≈ 1.0; see Supplementary Figures 8C, D for details). On the other hand, similar to the control condition, biphasic pulses with no interphase delay or a 100-μs delay produced the smallest response magnitude among most of the interphase-delay conditions tested (Figures 4E–H; see Supplementary Figures 8C–F for details). These demonstrate that the nonlinear facilitation with optimal interphase delays observed in the control condition is no longer significant under synaptic blockade.

We also examined the overall spatial profiles of the integrated response magnitude under synaptic blockade, following the same procedure used for the control condition. The spatial profiles for each stimulation condition are shown in Figures 4I, J (see Supplementary Figures 9A, B for data from individual slices). In contrast to the control condition, the Friedman test detected no significant differences across the stimulation conditions at either 10-μA/phase or 20-μA/phase amplitudes (*n* = 6, *p* > 0.053; Figures 4K, L). This result supports an idea that, under synaptic blockade, interphase delays neither result in nonlinear cumulative facilitation nor alter the overall spatial spread of the response.

We additionally analyzed the synaptically-mediated response component, which was obtained by subtracting the responses remained under synaptic blockade from those recorded under control conditions (Supplementary Figure 10A). The statistical analyses on the temporal summation of the subtracted response magnitude supported that, at 10-μA/phase amplitude, biphasic pulses with an interphase delay of 500, 600, or 1,000 μs elicited significantly larger excitation magnitudes than the cathodic pulses in the subsequent phase (*n* = 6, *p* < 0.012; see Supplementary Figure 10F), supporting that postsynaptic/trans-synaptic excitation contributes to the nonlinear cumulative facilitation.

## 4 Discussion

This study demonstrated that incorporating an optimally timed interphase delay into biphasic current pulses nonlinearly facilitates the stimulus-elicited neural activation in V1 of mouse brain slices. Notably, the facilitative effects of optimal interphase delays were shown to accumulate across multiple pulses, and pharmacological treatment confirmed that these effects involve trans-synaptic recruitment of neuronal populations. To our knowledge, this is the first *in-vitro* demonstration of the cumulative, interphase delay-dependent recruitment of cortical neuronal ensembles using millisecond-resolution VSD imaging. While the previous clinical study has reported a reduced detection threshold with longer interphase delays (Schmidt et al., 1996), direct physiological measurements in the cortex have been lacking. Our study bridges this gap by providing a mesoscopic-scale view of cortical activation patterns, thereby linking the psychophysical outcomes with population-level neural dynamics.

Biphasic current pulses are known as a standard form of stimulation for clinical neural stimulation, primarily for safety and long-term stability (Merrill et al., 2005; Cogan, 2008). This charge-balanced design is essential to prevent gradual shifts of the voltage at the electrode-tissue interface, and in turn electrochemical damage of electrodes and the biological tissues, which can be particularly critical during high-frequency stimulation (Cogan, 2008). However, this safety requirement creates a well-known safety–efficiency trade-off: biphasic pulses require larger current amplitude than cathodic monophasic pulses for eliciting neural excitation, as demonstrated in previous studies (Bromm and Frankenhaeuser, 1968; van den Honert and Mortimer, 1979; Miller et al., 2001; Rubinstein et al., 2002). Our observations of the initial depolarization in response to biphasic pulses (Figures 2B, C) align with classic electrophysiological theory and electromagnetic principles suggesting that incorporating interphase delays reduce the cancelation of cathodic-current-induced depolarization by the following anodic phase, thereby increasing the likelihood of action potential generation (van den Honert and Mortimer, 1979). It has been also known that lowering the current amplitude of anodic phase can reduce such a cancelation effect, thereby improving the efficacy of cathodic-phase current in eliciting membrane depolarization (Cogan, 2008). Our present results extend theses understandings by showing that the insertion of optimal interphase delays can actively contribute to the enhanced excitation under specific conditions.

Although the precise mechanism underlying the nonlinear facilitation produced by optimal interphase delays remains unclear, several observations from the present study constrain plausible explanations. First, an anodic monophasic pulse elicited an initial hyperpolarization followed by a spatially distributed depolarization that peaked near the stimulation site and persisted for tens of milliseconds (Figures 2A–E, h). This delayed depolarization was abolished under synaptic blockade (Supplementary Figures 2A–E, h), suggesting that the anodic phase can engage delayed trans-synaptic excitation in a subset of neurons or neuronal compartments. In contrast, cathodic pulses are considered to elicit depolarization in cellular membranes near the electrode tip, and also hyperpolarization at other membrane sites and/or later time window to form resistive and capacitive current loops in local extracellular space and in neurons (Note a possibility that such a hyperpolarization was masked by population action potential, and/or not visualized by VSD imaging due to its incoherent spatiotemporal distribution). A simple interpretation is that, when the interphase delay is near its optimal value, the delayed excitation triggered by the anodic phase arises in neural elements that are not strongly influenced by the hyperpolarization triggered by the cathodic phase in other elements—or that it emerges before such hyperpolarization-related attenuation becomes effective. On the other hand, shorter interphase delays would instead promote more direct cathodic–anodic counteraction. Given the cellular and compartmental heterogeneity of cortical neural circuits, not only this simple mechanism, but also multiple mechanisms may contribute to the nonlinear facilitation. Further computational and cell-type- or compartment-specific physiological studies will be required to delineate the circuit processes underlying this phenomenon.

While we did not systematically explore facilitative effects by interphase delay longer than 1,000 μs, it is considered that an excessively long interphase delay is neither physiologically optimal nor electrochemically safe. Physiologically, if the interphase delay becomes too long, the biphasic pulse train begins to approximate to alternative cathodic-anodic monophasic pulse train. Consequently, the interactions (including cancellation effects and/or facilitation effects) between the two phases are lost. Electrochemically, if the interphase delay is too long, the chemical intermediates can diffuse away from the electrode surface before they can be reversed by the anodic pulse (Cogan, 2008). This failure of reversal leads to irreversible reactions, which can cause electrode degradation and potential tissue damage (Merrill et al., 2005). The upper limit of the interphase delay inserted in the biphasic pulse is not electrochemically defined because this diffusion-limited timeframe is dependent on factors such as electrode material, geometry, and the local tissue environment (Merrill et al., 2005; Cogan, 2008). Therefore, the practical upper limit for the interphase delay is ultimately determined by whichever is shorter: the physiologically optimal duration that occurs maximal facilitation, or the absolute limit dictated by electrochemical safety. The latter should be clarified through long-term *in-vivo* animal studies.

The shift in the optimal interphase delay depending on stimulus amplitude (Figures 2, 3) highlights an interaction between the stimulation parameters and the dynamic state of cortical circuits, potentially due to threshold effects, synaptic integration dynamics, or circuit-level saturation. In the cortical response to suprachoroidal retinal stimulation, John et al. (2013) reported that the effect of inter-pulse delay is dependent on the phase duration, suggesting that the optimal interphase delay of biphasic pulses for ICMS may likewise vary with other pulse parameters. Such nonlinearity may underlie the variability in optimal stimulus parameters observed across cortical regions or behavioral contexts and warrants further investigation. In addition, while the optimal interphase delay at 10 μA/phase amplitude was identified, optimal interphase delays at lower and higher amplitudes were not determined. Also, at 20-μA/phase amplitude, it is considered that there is the optimal interphase delay because the biphasic pulses with 800- and 1,000-μs delays elicited significantly larger excitations than the cathodic monophasic pulse (which conceptually represents a biphasic pulse with an infinite interphase delay). However, the relative magnitude of the facilitation was less pronounced compared to that at 10 μA/phase amplitude (Figures 3E–H). This may suggest a saturation: if the initial cathodic phase already recruits a neuronal population near its saturation level, the additional facilitative effect provided by the optimal interphase delay may fail to substantially further enhance the excitation. In such cases, the interphase delay yielding the largest excitation may not be an optimal value, but rather correspond to the longest interphase delay (i.e., a plateau). Whether optimal interphase delays exist at amplitudes outside the 10–20 μA/phase range, and what their values might be, warrants further investigation through additional physiological experiments and computational simulations.

Despite the novel insights gained by the present results, this study has several limitations. First, the experiments were conducted in acute brain slice preparations. While the basic synaptic mechanisms are preserved, long-range horizontal and feedback connections are partially compromised (Thomson and Lamy, 2007; Schneider-Mizell et al., 2025; Weis et al., 2025). Due to the absence of inter-areal connections, it is difficult to assess communication between inter-cortical areas. Furthermore, although a horizontal propagation of excitation within layer II/III was observed in our VSD imaging (e.g., Figure 3), the excitation initiated in layer II/III could propagate vertically to infragranular layers and receive recurrent feedback (Burkhalter, 1989; Yuste et al., 1997; Thomson and Lamy, 2007). Such inter-layer interactions, particularly between layer II/III and layer V, serve as critical elements driving the horizontal propagation within layer II/III (Wester and Contreras, 2012). However, in our slice preparations, the connections between layer II/III and layer V could be severed by the slicing procedure, since we have not encountered a spontaneous up-state previously found in slice preparations (Sanchez-Vives and McCormick, 2000; Wester and Contreras, 2012). In addition, microstimulation of layer IV elicited relatively small excitation in layer V compared with layer II/III in our preparations, as reported in the previous study (Tanaka et al., 2019). Consequently, the observed facilitative effects may underestimate the full extent of interphase delay–mediated recruitment in intact cortical networks *in vivo*. Future work is needed to determine whether these facilitative effects occur *in-vivo* setting, potentially using different experimental methods (Miyamoto et al., 2017; Kordecka et al., 2025; Hughes et al., 2025). Second, although the study employed two stimulus amplitudes and a range of interphase delays, a more comprehensive parametric exploration could reveal finer-grained dependencies or interactions. Especially, the pulse trains used in this study were limited to a short train duration (11 pulses at 100 Hz, i.e. 110 ms). We did not capture neural depression caused during long pulse trains (e.g., 1–30 s), which has been extensively studied in the literature (Michelson et al., 2019; Sombeck et al., 2022; Hughes and Kozai, 2023; Kumaravelu and Grill, 2024; Hughes et al., 2025). Such a depression is thought to be related to perceptual fading of the ICMS-induced perceptions in humans (Schmidt et al., 1996; Hughes et al., 2021), and it is a critical component of the chronic stimulation response. Future work should investigate whether nonlinear facilitative effects of optimal interphase delays are maintained or modified during the prolonged stimulation patterns *in vivo*. Third, while VSD imaging provides excellent temporal resolution, it lacks cell-type specificity, and further studies using genetically encoded voltage indicators (Bando et al., 2019; Carandini et al., 2015) may reveal the contributions of specific neuronal populations. Moreover, computational modeling approaches that incorporate realistic synaptic dynamics and circuit architecture may provide further insights into the biophysical basis of the observed phenomena.

From a clinical perspective, the present findings offer important implications for the design of intracortical visual prostheses, providing new evidence that optimally timed cathodic-anodic interaction actively modulates neural circuit activation—particularly through cumulative trans-synaptic mechanisms. Optimizing interphase delay could improve the efficacy of percept generation by maximizing neural recruitment while minimizing charge delivery in intracortical neural prostheses.

## Data Availability

The original contributions presented in this study are included in this article/Supplementary material, further inquiries can be directed to the corresponding author.
