# Supplementary material for: Optimal interphase delay in biphasic current pulses facilitates neural circuit activation induced by microstimulation in the mouse visual cortex
Source: Front Neurosci. 2026 Jan 12;19:1710221. doi: 10.3389/fnins.2025.1710221 (PMC12833007; doi:10.3389/fnins.2025.1710221)
Supplement: Supplementary file 1 [file Data_Sheet_1.pdf]

frontiers

Supplementary Figures

Supplementary Figure 1

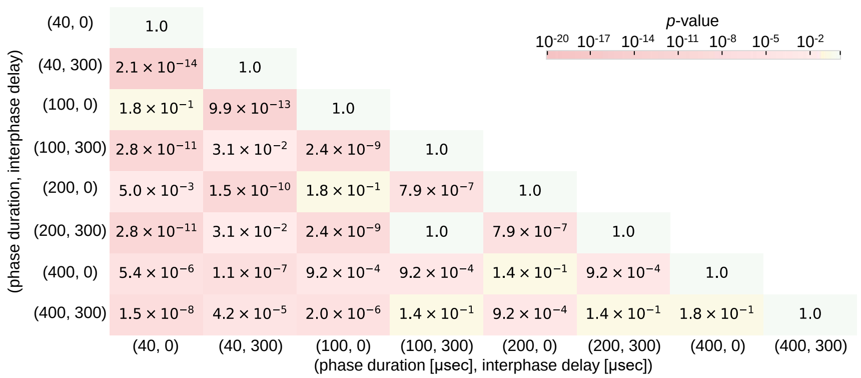

Supplementary Figure 1. Detailed statistical results for Figure 1*G*. This matrix displays the *p*-values obtained from Conover's post-hoc test shown in Figure 1*G*. The colormap indicates the level of statistical significance for each pairwise comparison; red-shaded cells indicate $p < 0.05$.

Supplementary Figure 2

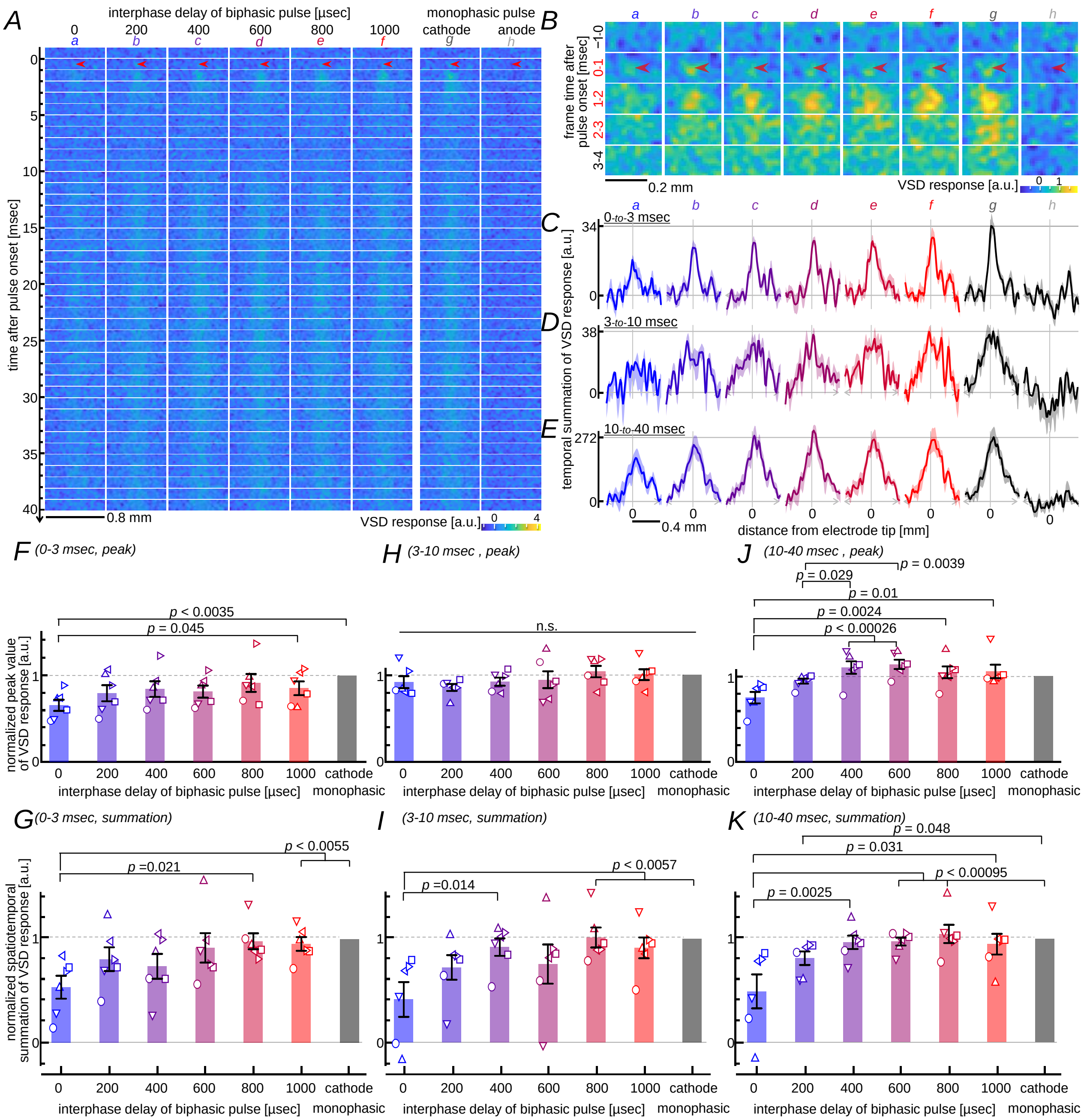

Supplementary Figure 2. Comparison of neural excitation elicited by single-pulse stimuli under synaptic blockade. From six out of the twelve slices used in the experiment shown in Figure 2, VSD responses to monophasic and biphasic current pulses were recorded in the presence of the glutamate receptor antagonists D-AP5 and DNQX. The VSD responses were averaged across the six slices. The overall figure format and analysis procedures are identical to those used in Figure 2. In panels *F*-*K*, specific *p*-values are shown instead of asterisks.

Supplementary Figure 3

(control, 0-3 msec)

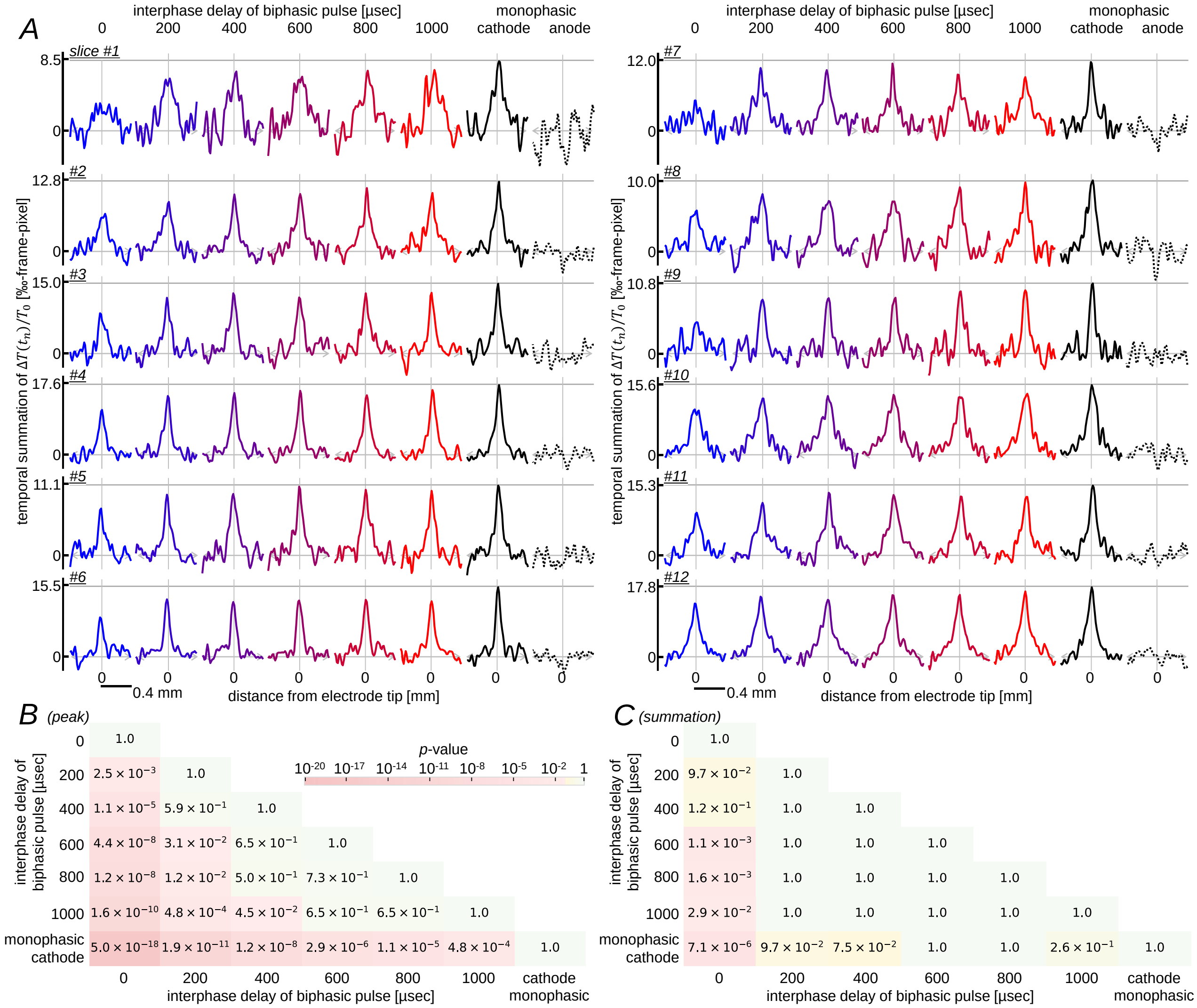

Supplementary Figure 3. Detailed analyses of the spatial profiles of temporally summated responses for 0–3 msec after pulse onset. This figure corresponds to Figure 2*C* and 2*F*-*G*. A) Spatial profiles of the temporally summated VSD responses for each of the twelve individual slices (labeled #1–#12). These traces represent the individual data used to calculate the mean profiles shown in Figure 2*C*. *B*-*C*) Matrices displaying the detailed *p*-values from Conover's post-hoc tests for the statistical comparisons shown in Figure 2*F*-*G*. The colormap indicates the level of statistical significance; red-shaded cells indicate $p < 0.05$.

Supplementary Figure 4

(control, 3-10 msec)

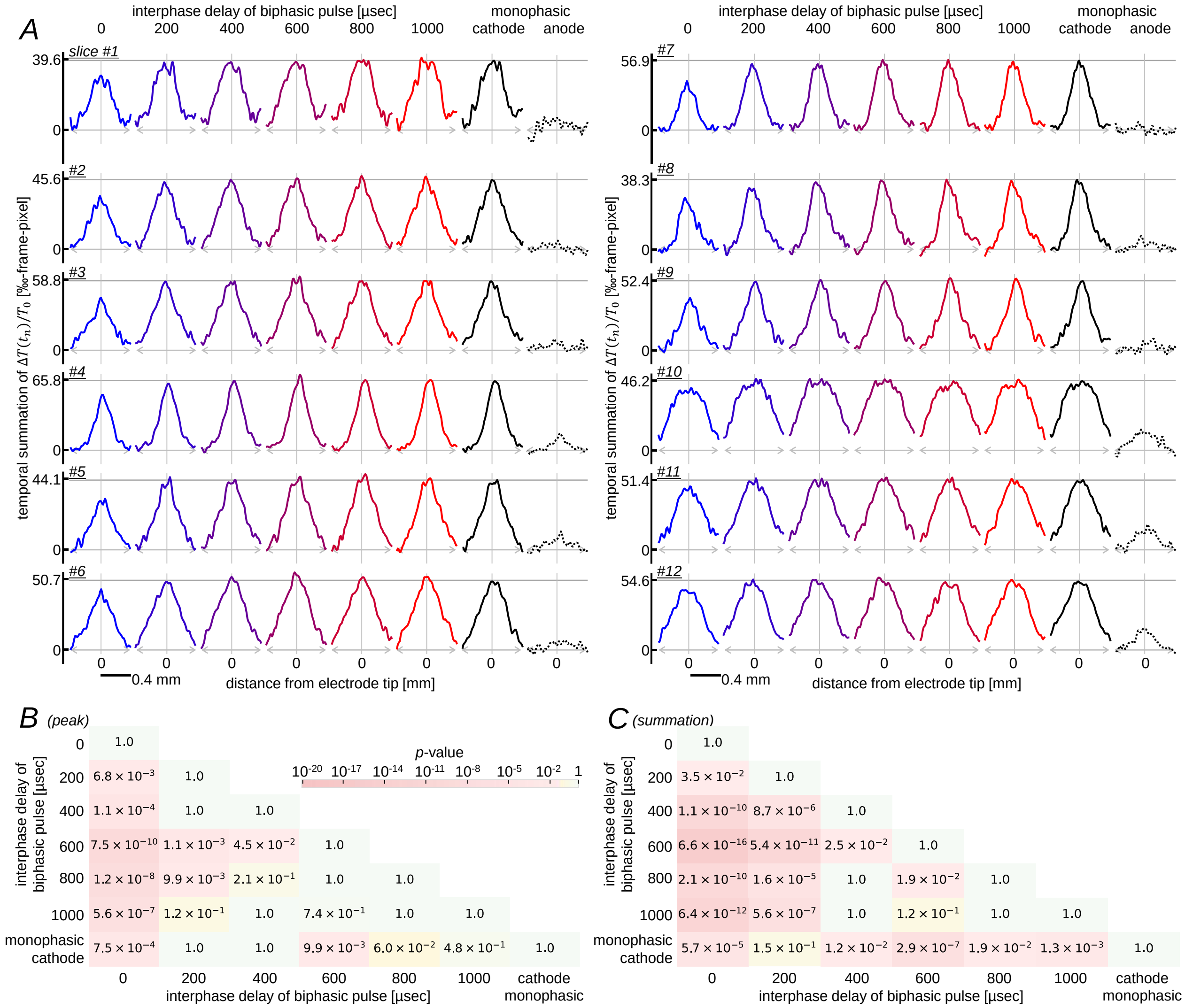

Supplementary Figure 4. Detailed analyses of the spatial profiles of temporally summated responses for 3-10 msec after pulse onset. This figure corresponds to Figure 2*D* and *H-I*. The overall figure format and analysis procedures are identical to those in Supplementary Figure 3.

Supplementary Figure 5
(control, 10-40 msec)

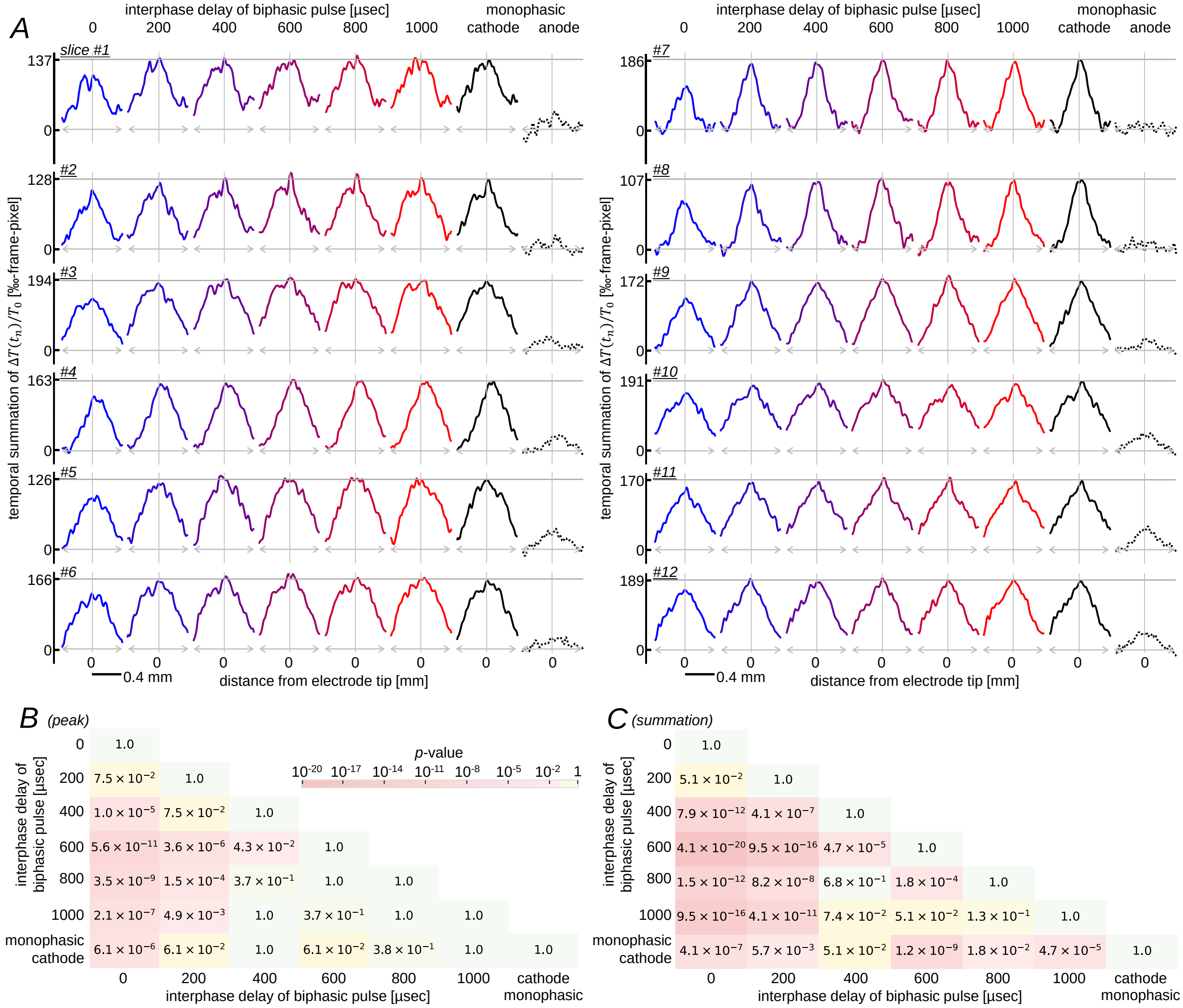

Supplementary Figure 5. Detailed analyses of the spatial profiles of temporally summated responses for 10-40 msec after the pulse onset. This figure corresponds to Figure 2*E* and *J*-*K*). The overall figure format and analysis procedures are identical to those in Supplementary Figure 3.

Supplementary Figure 6

(control, spatiotemporal summation)

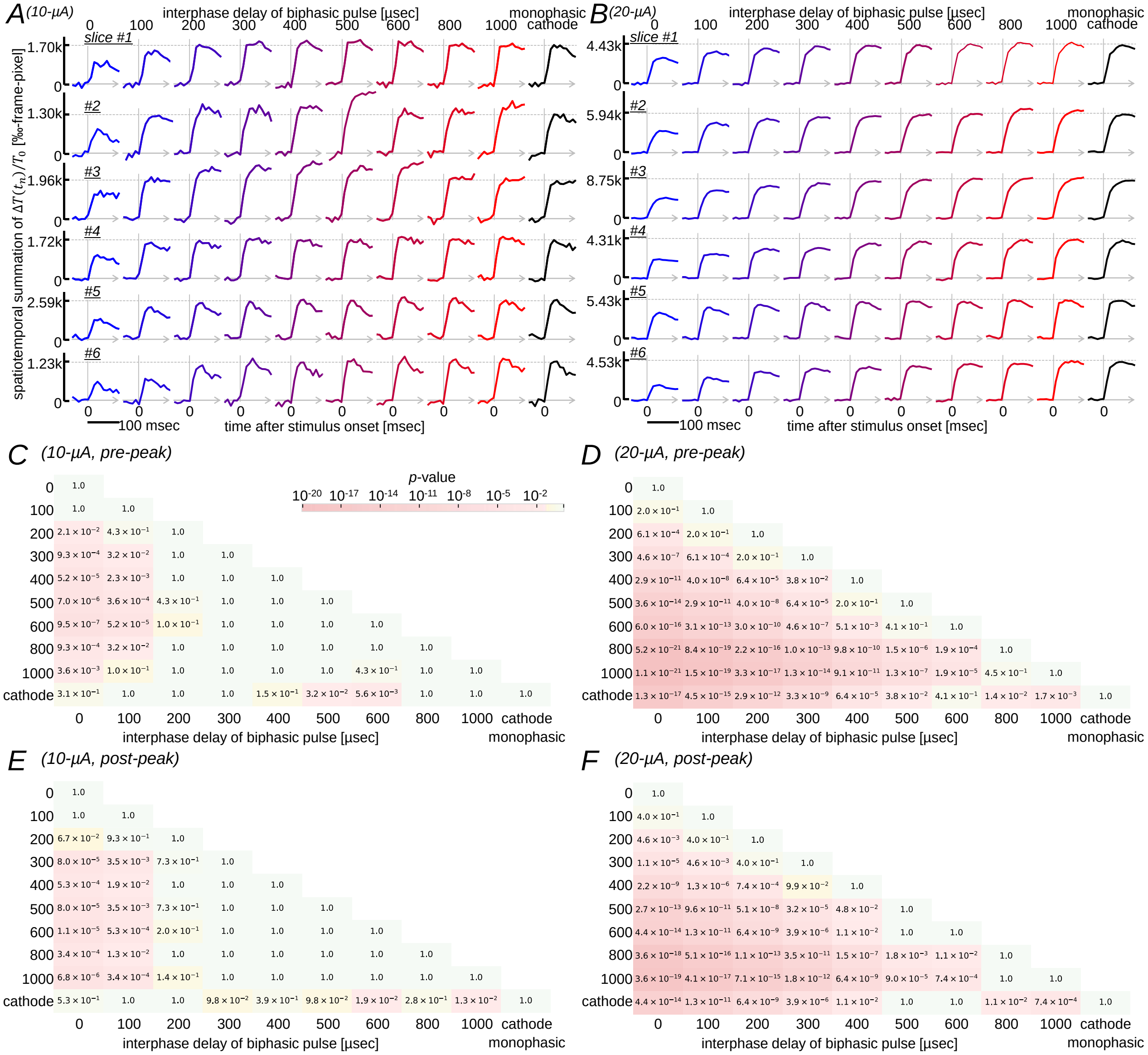

Supplementary Figure 6. Detailed analyses of the time courses of response magnitudes during pulse-train stimulation. This figure corresponds to Figure 3*C-H*. *A-B*) Time courses of the response magnitude for each of the six individual slices (labeled #1–#6). These traces represent the individual data used to calculate the mean time-courses shown in Figure 3*C-D*. *C-F*) Matrices displaying the detailed *p*-values from Conover's post-hoc tests for the statistical comparisons shown in Figure 3*E-H*. The colormap indicates the level of statistical significance; red-shaded cells indicate $p < 0.05$.

Supplementary Figure 7

(control, half-energy width)

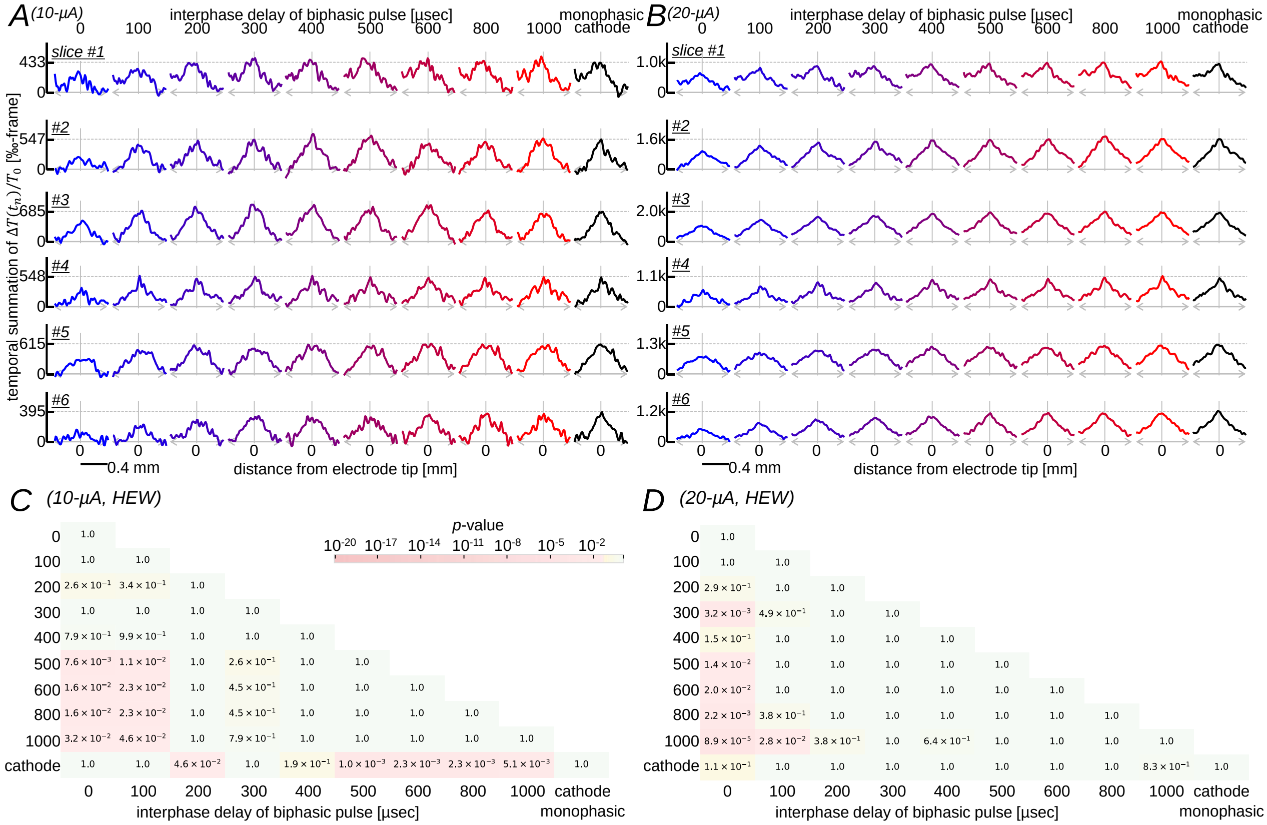

Supplementary Figure 7. Detailed analysis of the overall spatial profiles of temporally summated responses during pulse-train stimulation. This figure corresponds to Figure 3*I-L*. *A-B*) Overall spatial profiles of temporally summated VSD responses for each of the six individual slices (labeled #1–#6). These traces represent the individual data used to calculate the mean profiles shown in Figure 3*I-J*. *C-D*) Matrices displaying the detailed *p*-values from Conover's post-hoc tests for the statistical comparisons shown in Figure 3*K-L*. The colormap indicates the level of statistical significance; red-shaded cells indicate $p < 0.05$.

Supplementary Figure 8

(+D-AP5&DNQX, spatiotemporal summation)

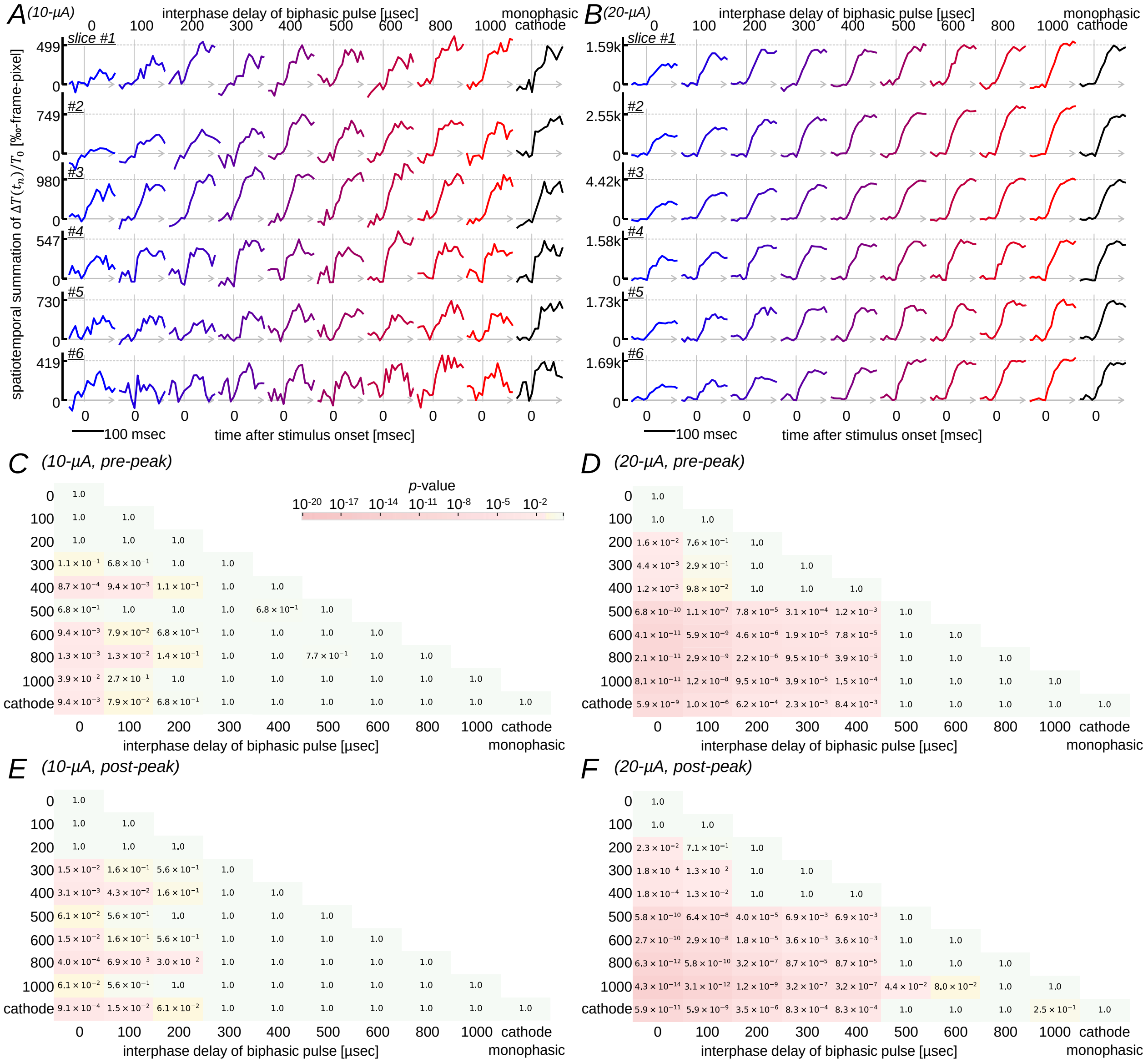

Supplementary Figure 8. Detailed analyses of the time courses of response magnitudes during pulse-train stimulation under synaptic blockade. This figure corresponds to Figure 4*C-H*. The overall figure format and analysis procedures are identical to those in Supplementary Figure 6.

Supplementary Figure 9

(+D-AP5&DNQX, half-energy width)

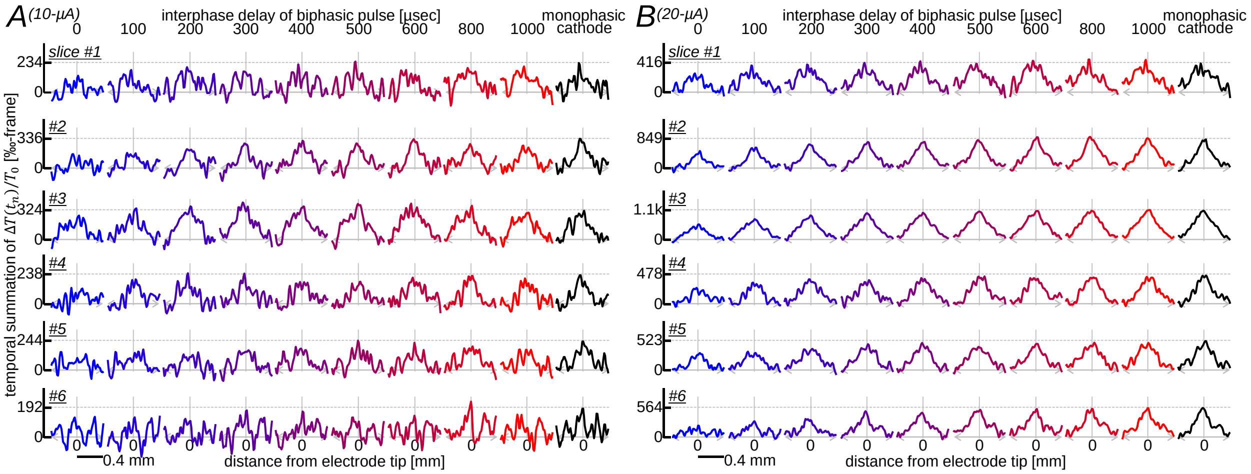

C (10-µA, HEW)

n.s. (n=6, p=0.053, Friedman test)

D (20-µA, HEW)

n.s. (n=6, p=0.37, Friedman test)

Supplementary Figure 9. Detailed analyses of the overall spatial profiles of temporally summated responses during pulse-train stimulation under synaptic blockade. This figure corresponds to Figure 4*I*-*L*. The overall figure format and analysis procedures are identical to those in Supplementary Figure 7, except that panels *C*-*D* in this figure describes only results of the Friedman tests in text.

Supplementary Figure 10

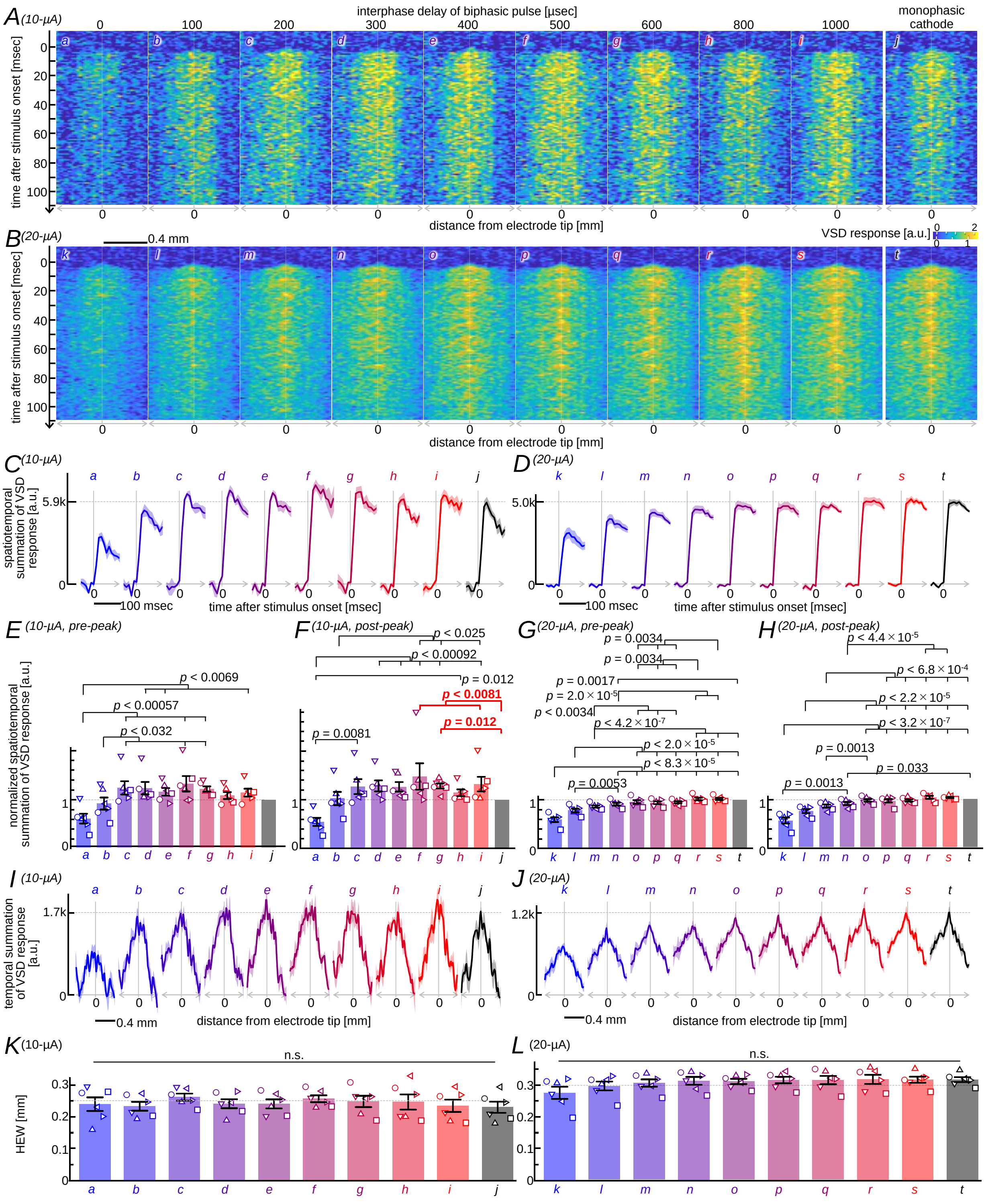

Supplementary Figure 10. Comparison of trans-synaptic neural excitation elicited by pulse-train stimulation. To extract the trans-synaptic components of the VSD responses, the VSD signals recorded under synaptic blockade (Figure 4) were subtracted from those recorded under control conditions (Figure 3). The overall figure format and analysis procedures are identical to those used in Figure 3. In panels *E*-*H*, specific *p*-values are indicated instead of asterisks.
