# Supplementary material for: Optimal interphase delay in biphasic current pulses facilitates neural circuit activation induced by microstimulation in the mouse visual cortex
Source: Front Neurosci. 2026 Jan 12;19:1710221. doi: 10.3389/fnins.2025.1710221 (PMC12833007; doi:10.3389/fnins.2025.1710221)
Supplement: Supplementary file 2 [file Data_Sheet_2.pdf]

Supplementary Methods

1 Ethics approval statement

All animal care and experimental procedures were approved by the Animal Experiment Committee of Mie University (approval no. 2021-19-Sai1-Hen1), and conducted in conformity with the Guidelines for Proper Conduct of Animal Experiments by the Science Council of Japan.

2 Mice and housing

C57BL/6J mice (4-7 weeks old at the time of experiments, female, n = 24) were used. Mice were obtained at 3-4 weeks of age from CLEA Japan, Inc. (Tokyo, Japan), and housed individually or in groups of up to three with free access to food and water under controlled conditions (22 ± 1 °C, 55 ± 5 % humidity, 12 h light/dark cycle; lights on at 07:00) in Experimental Animal Facilities of Mie University until the experiments.

3 Preparation of mouse brain slices for VSD imaging

Mice were euthanized by isoflurane overdose (≥5% in ambient air) in an induction chamber until a loss of reflexes and cessation of respiration were confirmed, followed by decapitation as a secondary method. The cerebrum was rapidly isolated, and submerged for 5 minutes in ice-cold sucrose-based artificial cerebrospinal fluid (aCSF) aerated with 95% O_2 + 5% CO_2. The sucrose-based aCSF was prepared to contain (in mM): 222 sucrose, 27 $NaHCO_3$, 1.5 NaH_2PO_4, 2.6 KCl, 7.0 $MgSO_4$, and 0.5 $CaCl_2$ (pH 7.4 under aeration).

Coronal slices (300 µm thick) including the primary visual cortex (V1) were prepared in this ice-cold, aerated solution using a vibratome tissue slicer (VT-1000S; Leica Microsystems, Nussloch, Germany). The slices were then incubated in a submerged chamber containing normal aCSF aerated with 95% O_2 + 5% CO_2, with the aCSF gently stirred using a magnetic stirrer (RCH-3L; EYELA, Tokyo, Japan) for 60 minutes at room temperature. The normal aCSF contains (in mM): 126 NaCl, 26 $NaHCO_3$, 1.14 NaH_2PO_4, 3 KCl, 1.0 $MgSO_4$, 3 $CaCl_2$, and 10 D-glucose (pH 7.4 under aeration).

Following incubation, tissue slices were transferred to a staining dish, and 800 µl of normal aCSF, containing the absorption-type oxonol voltage-sensitive dye (VSD; 31.25 µg/ml; NK3630, 14-1C; Nippon Kankoh-Shikiso Kenkyusho, Okayama, Japan; originally synthesized by Hildesheim and Grinvald as RH482) was applied onto the tissue surface. The slices were then stored in a humidified incubator (35–37 °C) for 60 minutes with continuous supplying 95% O_2 + 5% CO_2.

After staining, the slices were washed three times with normal aCSF to remove residual dye and incubated for an additional 15 minutes in the same humidified environment. Finally, the slices were transferred to the submerged chamber and allowed to recover for at least 10 minutes before use.

4 Experimental setup

The VSD imaging setup is illustrated in S-M-Figure 1. The tissue slice in a recording chamber was illuminated from below through a cover glass with spatially uniform collimated light provided by

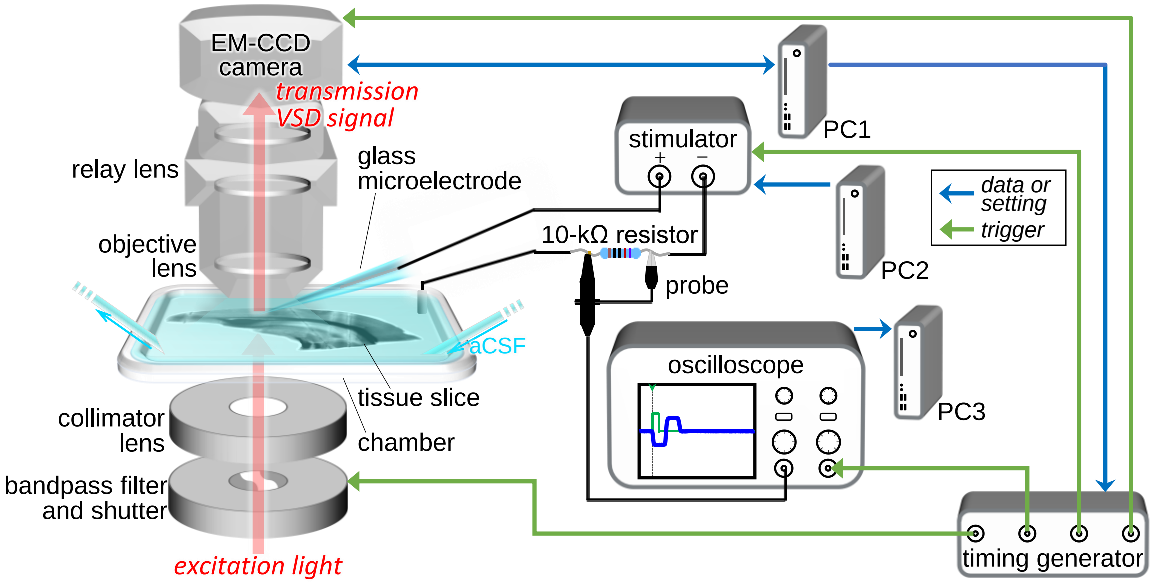

S-M-Figure 1. Experimental setup for VSD imaging. Green arrows indicate the flow of trigger pulses sent from the pulse generator. Blue arrows indicate the transmission and reception of recorded data and detailed setup information. Red arrows indicate the flow of optical signal.

a halogen lamp (100 W; U-LH 100 IR; Olympus, Tokyo, Japan) via a custom-made shutter equipped with a bandpass filter (694 ± 10 nm; O.D. 4.0 TECHSPEC; Edmund Optics, NJ, U.S.A.). The transmitted light image of the tissue slice was captured by an electron multiplying charge-coupled device (EM-CCD) camera (sensor size 8.2 × 8.2 mm, 512 × 512 pixels; iXon 3 DU-897; Andor Technology, Belfast, U.K.) through a 10× water-immersion objective lens (0.3 NA; UMPlanFI; Olympus) and a neutral density (ND) filter (10% transmittance; Edmund Optics). To achieve a high frame-sampling rate (i.e. 1000 fps), a limited region within V1 (referred to as the local field of view, 'LFV'; shown in S-M-Figure 2) was acquired employing the crop mode of the EM-CCD camera. The LFV corresponded to 96 × 512 pixels on the EM-CCD sensor. These pixels were binned using an 8-×8-pixel window, resulting in an effective sampling resolution of 12 × 64 pixels at 1000 fps. During the experiment, aCSF aerated with 95% O_2 + 5% CO_2 was continuously perfused into the recording chamber.

All biphasic and monophasic current pulses were generated using a desk-top stimulator (STG4002, MultiChannel Systems, Reutlingen, Germany). For stimulation, a glass microelectrode with an open tip diameter of approximately 6 μm was used, filled with normal aCSF and contained an internal Ag/AgCl wire connected to the active node of the stimulator. The electrode tip was inserted into layer II/III of V1 at a depth of 100-150 μm from the cut surface. An Ag/AgCl pellet positioned at a distant location in the chamber served as the return electrode. Pulse waveforms and amplitudes were monitored by inserting a 10-kΩ resistor in series between the return electrode and the current return node of the stimulator, and recording the voltage across this resistor with an oscilloscope (TDS2024B; Tektronix, Beaverton, U.S.A.).

All devices (EM-CCD camera, shutter, stimulator, and oscilloscope) were synchronized using a timing pulse generator (Master-8; A.M.P.I., Jerusalem, Israel), as illustrated in S-M-Figure 1. The timing generator sent trigger pulses to each device according to pre-programmed time intervals. As an initial state, the shutter remained closed to minimize photobleaching when VSD signals were not being recorded. The recording sequence was initiated by trigger pulses sent to the EM-CCD camera (to start

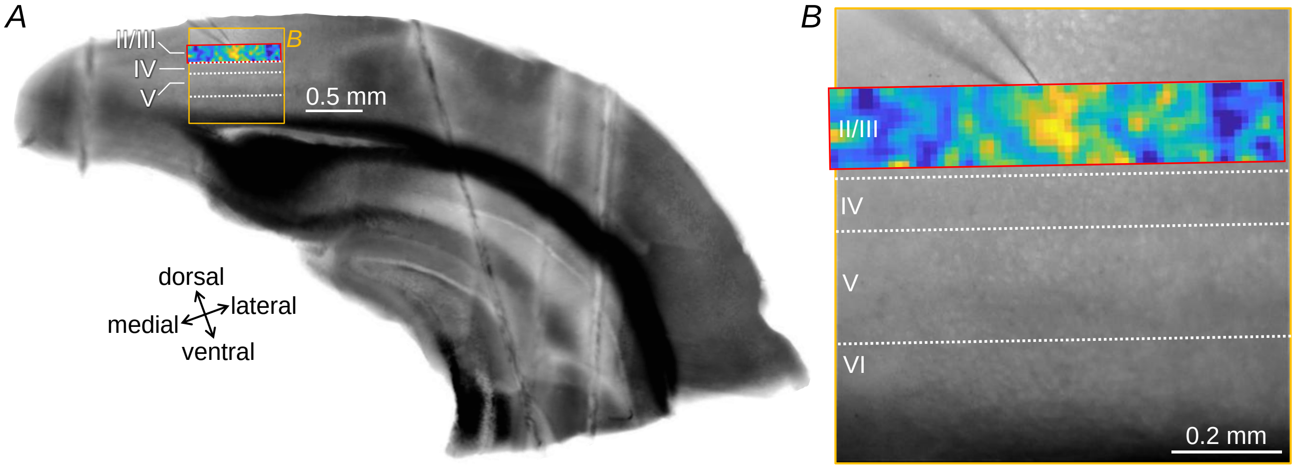

S-M-Figure 2. A) Infrared images of a cerebral slice at lower magnification. B) High magnification view of the area indicated by the yellow rectangle in A. The dotted horizontal lines indicate approximate borders between cortical layers based on the somal shape, size, density, and distance from the cortical surface. The overlaid color image is a sample image frame from VSD signals obtained in this experiment, highlighting the LFV in the cerebral slice.

a 1500-msec acquisition) and to the shutter (to open). At 300 msec after the onset of recording, the electrical stimulation was delivered and the waveform of stimulus current pulse was recorded. Finally, at 2000 msec after the recording onset (i.e., 500 msec after the end of acquisition), the shutter was closed.

This sequence was repeated at intervals of 10-17 sec, with the stimulation condition systematically switched among a set of predefined conditions. For each condition, recordings were repeated 15-180 times, and responses were averaged across repetitions. The number of repetitions varied across slices but remained constant across all stimulation conditions, including the no-stimulation condition, within each individual slice.

5 Pharmacological treatments

To block excitatory synaptic transmission in the cortex, the superfusate was supplemented with D-2-amino-5-phosphonovaleric acid (D-AP5; 20 µM; Cayman Chemical Co., Ann Arbor, U.S.A.), an NMDA-type glutamate receptor antagonist, and 6,7-dinitroquinoxaline-2,3-dione (DNQX; 10 µM; Cayman Chemical Co., Ann Arbor, U.S.A.), a non-NMDA-type glutamate receptor antagonist. Recording was paused during the exchange of the superfusate and resumed once the antagonist-containing aCSF reached the recording chamber. VSD signals obtained within the first 10 min after resumption were excluded from the analysis to ensure complete wash-in of the antagonists. The recording under this condition was also repeated at intervals of 10–17 sec intervals, with stimulation conditions switched among predefined settings. The number of repetitions was also kept constant across all stimulation conditions within each individual tissue slice and matched that used under the control condition.

6 Signal pre-processing

All image processing was performed with MATLAB (ver. R2023a; MathWorks, Natick, U.S.A.) The acquired VSD signals were pre-processed for quantitative analysis following the steps outlined in the flowchart shown in S-M-Figure 3.

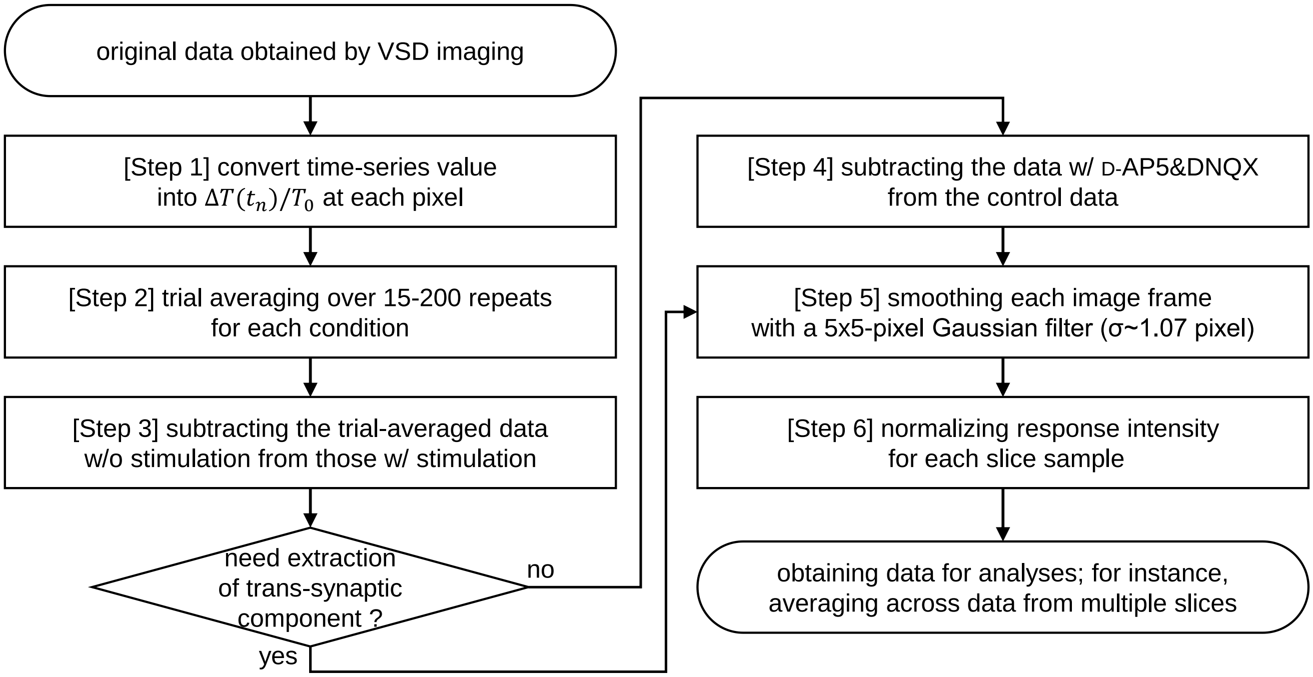

S-M-Figure 3. Flowchart of pre-processing of VSD signals

Step 1: The relative change in optical intensity was quantified as $\Delta T(t_n)/T_0$, where T_0 denotes the average transmission intensity before stimulation and $\Delta T(t_n)$ represents the change in transmission intensity from T_0 at the discrete time point t_n. This calculation was performed at each pixel of the captured image series. T_0 was defined as the mean intensity over the 22-10 msec period before stimulation onset at each pixel. Positive and negative values of $\Delta T(t_n)/T_0$ correspond to membrane depolarization and hyperpolarization, respectively.

Step 2: VSD signals from all repeated trials (15–180 per condition) were averaged to improve the signal-to-noise ratio. This averaging was performed for each pixel and each frame.

Step 3: The VSD signals in no-stimulation trials were subtracted from those obtained under stimulation, for each pixel and each frame.

Following pre-processing (Steps 1-3), the resulting VSD signals (both control and synaptic blockade) were routed into two analysis pipelines. In the first pipeline, used for analysis of VSD signals under either control or synaptic blockade condition, Step 4 was skipped and Step 5 (described below) was applied directly. In the second pipeline, used to extract trans-synaptic response component (i.e., the component suppressed by D-AP5 and DNQX), Step 4 involved subtracting the VSD signals obtained under the synaptic blockade condition from those obtained under control condition. This subtraction was performed for each pixel and each frame.

Step 5: Each VSD image was spatially filtered using a Gaussian filter (5 x 5-pixel, s.d. ~ 1.07) to improve the signal-to-noise ratio. The filter size was determined based on an analysis results of the filter's effects on the VSD signals (see Section 7).

Step 6: To enable averaging across slices, the VSD signals were normalized to compensate for inter-slice variations in response amplitude. The VSD signal intensity at each pixel was divided by a

reference amplitude calculated for each slice. The reference amplitude was defined as the mean VSD signal amplitude (averaged across all pixels and over 0-100 msec after pulse onset) elicited by the cathodic monophasic pulse. When this pulse was not applied, the response elicited by a cathodic-first biphasic pulse (with no interphase delay and with 200-μsec phase duration) was used instead.

Finally, the normalized VSD signals were used for subsequent averaging across six or twelve tissue slices.

7 Effects of spatial filtering on the time course of VSD responses

To determine the proper size of the Gaussian spatial filter used in the present analyses, we first performed a quantitative evaluation using simulated VSD signals, as illustrated in S-M-Figure 4. The simulated signals were generated by combining two components: a realistic random noise component and a noise-free response signal, as schematically shown in panel *A*.

To generate random noise resembling that of actual VSD signals, we analyzed the spatial and temporal characteristics of baseline VSD noise recorded from a single tissue slice during a 200-msec pre-stimulus period (from −220 to −20 msec relative to stimulation onset), across all pixels. This analysis employed amplitude spectra and autocorrelation functions in space and time domains (panel *B*, black traces and plots). Spatially, the amplitude spectrum (upper-left subpanel in *B*) showed relative dominance of high spatial frequency components, and the autocorrelation plot (upper-right subpanel in *B*) exhibiting a negative peak at a lag of 1 pixel and a decay to near zero at larger lags (>2 pixels). Temporally, neither the amplitude spectrum nor the autocorrelation plot (lower subpanels in *B*), indicating the absence of structured temporal periodicity.

Based on these characteristics, random noise was generated independently for each time frame. First, a one-dimensional (1D) double-Gaussian function was fitted to the observed spatial autocorrelation profile (*B*, upper-right subpanel, black traces and plots in the inset). This fitted function was then expanded into a two-dimensional (2D) double-Gaussian function, which served as a 2D spatial kernel. Next, a random noise pattern with values uniformly distributed between -1 and 1 was generated and spatially convolved with this kernel, resulting in a realistic spatial noise pattern. The processing scheme is illustrated in the upper-left in panel *A*. The spatial noise patterns generated for each frame were then combined to form a time-series noise image. This time-series noise image closely reproduced the spatial and temporal characteristics of the recorded VSD noise, as confirmed by the red traces and plots in panel *B*.

To construct the noise-free response signal, 1D Gaussian functions were fitted to the longitudinal spatial profiles of recorded VSD responses for each time frame during the 0-9 msec period following pulse onset (panel *C*; experimental data with black plots, fitted functions with red lines). From these fitted 1D functions, corresponding 2D Gaussian functions were generated and used as noise-free simulated response images. The processing scheme for each time frame is illustrated in the lower-left in panel *A*. The simulated spatial response images for each frame were then combined to form a time-series response image. The processing scheme is illustrated in the lower-left in panel *A*, as shown in

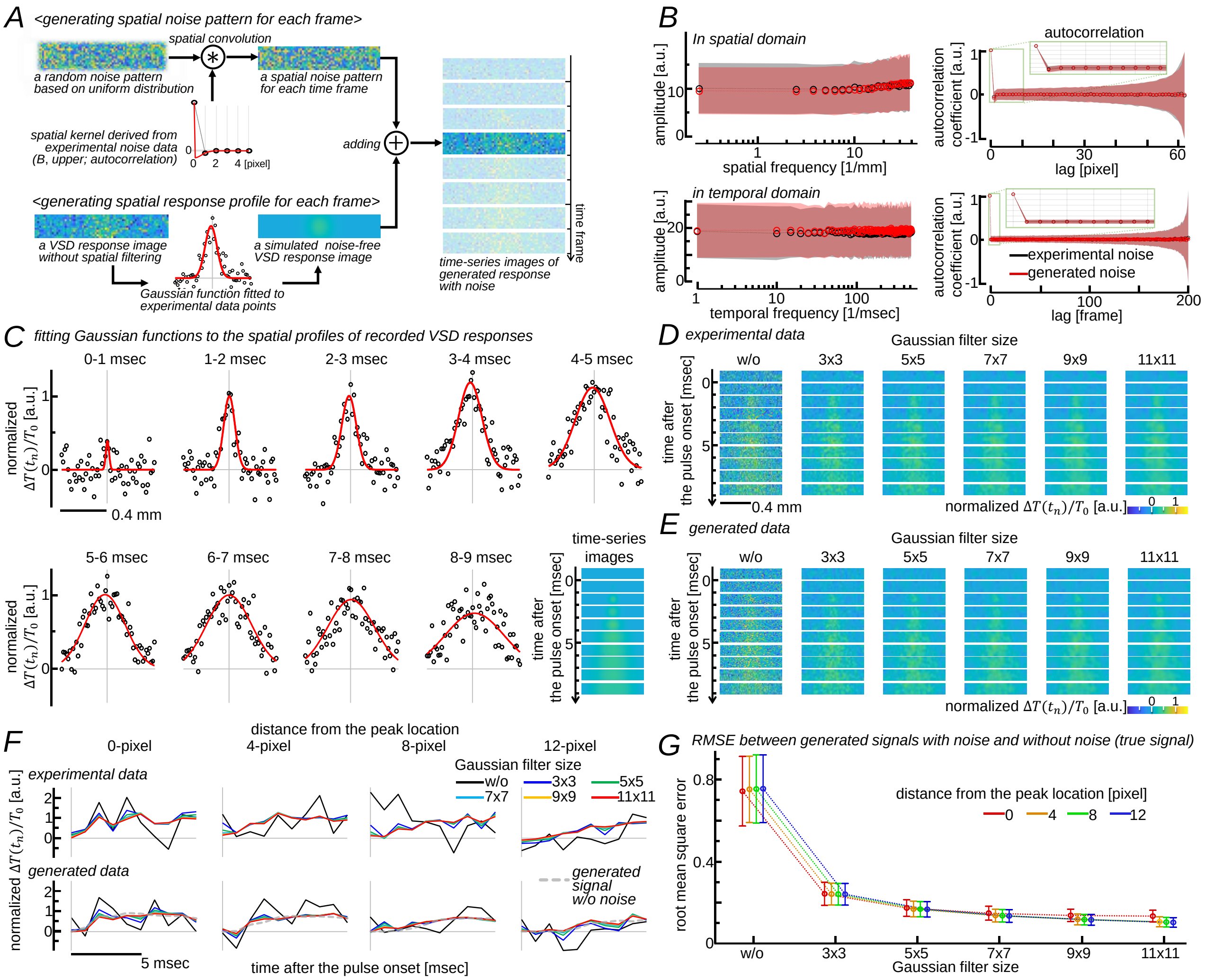

S-M-Figure 4. Effects of Gaussian filtering on simulated signals. A) Schematic of the method used to generate simulated signals. B) Comparison of noise characteristics on actual VSD signals (black) and the generated (simulated) random noise (red). Amplitude spectrum (left panels) and autocorrelation (right panels) along the spatial axis (top panels) and the temporal axis (bottom panels) were shown. In these plots, markers represent the mean, and shadings represent the s.d. These statistics were calculated across all pixels along the short axis and all frames (for the top panels), and across all pixels (for the bottom panels), respectively. C) Fitting result for generating noise-free signal. The actual averaged VSD signal (black circles) was fitted with a one-dimensional (1D) Gaussian function (red line). The equation for this fitting function is shown in panel A (red text). The time-series images in the bottom-left of C show the resulting noise-free signal used for the simulation, which was a 2D Gaussian function constructed from the fitting result of the 1D Gaussian function. D) Time-series images of the averaged actual VSD signals, across six tissue slices. E) Time-series images of the averaged simulated signals, across six trials. In D and E, the signals were Gaussian-filtered with each filter size prior to averaging. F) Time courses of the actual VSD signals from D (upper) and the simulated signals from E (bottom), extracted at 0, 4, 8, and 12 pixels from the peak signal location. The dotted line in the bottom panel indicates the time course of the simulated signals without random noise. G) Root-mean-square-error (RMSE) between the simulated VSD signals (averaged across six trials) and the simulated signals without random noise, extracted at 0, 4, 8, and 12 pixels from the peak signal location. Markers represent the mean of RMSE and error bars represent the s.d., across 1000 repetitions of this 6-trial-averaging simulation.

the bottom-right in *C*. Subsequently, by adding the generated spatial noise patterns to these simulated noise-free VSD response images, simulated noisy VSD response images were produced, as schematically illustrated in the right in panel *A*.

To replicate the averaging across different slices performed for the experimental data, six independent simulated datasets were generated. Both the recorded VSD images from six slices and the simulated noisy VSD images generated for six independent datasets were processed using Gaussian filters of varying sizes: 3×3-pixel (s.d. ~ 0.79 pixel), 5×5 (s.d. ~ 1.07 pixel), 7×7 (s.d. ~ 1.28 pixel), 9×9 (s.d. ~ 1.42 pixel), and 11×11 pixels (s.d. ~ 1.51 pixel). These filtered signals were subsequently averaged across the six slices or six simulated datasets, respectively.

S-M-Figure 4*D-E* shows time-series images of the averaged VSD responses processed with different filter sizes, obtained from actual experiments (*D*) and obtained from the simulation (*E*). Although the unfiltered images (leftmost panels) exhibited substantial noise, application of the Gaussian filter markedly improved the signal-to-noise ratio. However, the smoothing effect appeared to saturate for filter sizes of 5 × 5 pixels or larger.

S-M-Figure 4*F* shows time courses of the averaged VSD responses extracted from four spatial locations (0, 4, 8, and 12 pixels from the peak response position). At all these locations, increasing the Gaussian filter size resulted in progressively stronger smoothing. For the simulated VSD responses (lower subpanels of *F*), larger filter sizes reduced the discrepancy between noise-free responses (gray dashed lines) and noisy responses (colored solid lines). To quantitatively assess this effect, root-mean-square error (RMSE) between the simulated noise-free responses and the corresponding noisy responses with and without filtering was calculated (S-M-Figure 4*G*). All Gaussian filters reduced the RMSE to less than 50% of the unfiltered value; however, further reductions nearly saturated for filter size of 5 × 5 pixels and above. Based on these results, a 5 × 5-pixel Gaussian filter was adopted for all offline processing in the present study.

8 Specific Analyses

8.1 Spatial summation

To reveal the temporal dynamics of the VSD response, we computed the time course of spatial summation. Spatial summation was defined as the summated response amplitude across all pixels, for each image frame.

8.2 Temporal summation

To characterize the spatial dynamics of VSD responses, we computed the spatial profile of temporal summation. Temporal summation was defined as the summated response amplitude across all pixels along the short axis and over a specified time window, for each position along the longitudinal axis.

8.3 Spatiotemporal summation

To quantify the overall magnitudes of VSD responses, we computed the spatiotemporal summation, defined as the summated response amplitude across all pixels and over the specified time window.

8.4 Space-time plot

To visualize the spatiotemporal dynamics of VSD responses, we constructed space-time plots. These plots were generated by sequentially stacking the longitudinal-axis spatial profiles (averaged

along the short axis) for each frame. The response amplitude was displayed in pseudo color as a function of time (vertical axis) and spatial pixel position (horizontal axis).

8.5 Half-energy widths

To quantify the spatial spread of VSD responses without assuming a Gaussian profile, we calculated the half-energy width (HEW). First, a spatial profile of temporal summation for 0-110 msec after pulse onset (see Section 8.2) was obtained. This profile was then rectified and symmetrically integrated from the center (the stimulation site) to the peripheries. The HEW was defined as twice the distance from the stimulation site at which the cumulative integral reached 50 % of the total response amplitude.

9 Statistical Analysis

Statistical analyses were performed with Python (ver. 3.10.0; Python Software Foundation, Wilmington, U.S.A.) using data exported from MATLAB in CSV file format. To determine whether significant differences existed among stimulation conditions in stimulus charge and VSD response outcome measures, non-parametric comparisons were conducted using the Friedman test. In the statistical analyses of VSD response measures, the responses elicited by the anodic monophasic pulses were excluded (Figure 2*A*–*E*, *h*). When the Friedman test indicates a significant difference ($p < 0.05$), Conover's post-hoc test with Holm–Bonferroni correction was subsequently performed to identify specific differences between conditions. Values used for statistical analyses are presented in bar plots as mean ± standard error of the mean (s.e.m.), and p-values obtained from Conover's post-hoc test are presented in heatmaps.

10 The dataset and the used waveform of current pulses

For the present study, four distinct datasets were acquired using a total of 24 tissue slices. For each dataset, the experiments were repeated until data from six distinct tissue slices were obtained.

In the first dataset, we applied only cathodic-first biphasic pulses to six tissue slices. The phase duration of the biphasic pulses was either 40, 100, 200, or 400 µsec. The stimulus-charge was set to 2 nC/phase, and the current amplitude was modified depending on each phase duration (i.e. when the phase duration is 200 µsec, the current amplitude is 10 µA). In these pulses, the interphase delay was set to either 0 or 300 µsec. The recorded traces of the current pulses are shown in Figure 1*B*. There was no significant difference in the charge of the cathodic phase among the eight biphasic pulses ($n = 6$, Friedman test, $\chi^2(7) = 11.5$, $p = 0.12$; Figure 1*C*). The VSD signals were obtained under only the control condition.

In the second and third datasets, we applied a cathodic monophasic pulse, an anodic monophasic pulse, and cathodic-first biphasic pulses with different interphase delays to six distinct tissue slices in each dataset. The parameters of the monophasic pulses were set to ±10 µA in current amplitude and 200 µsec in pulse duration. The parameters of the biphasic pulses were set to 10 µA/phase in current amplitude, and 200 µsec in phase duration. The interphase delay was set to either 0, 200, 400, 600, 800, or 1000 µsec. The recorded traces of the current pulses are shown in S-M-Figure 5*A*. There were significant differences in the stimulus charge among the eight pulses ($n = 12$, Friedman test, $\chi^2(7) = 16.2$, $p = 0.023$; S-M-Figure 5*B*). Conover's post-hoc test revealed that the charge of the biphasic pulse with no interphase delay was significantly smaller than some of the other pulses (S-M-Figure 5*C*).

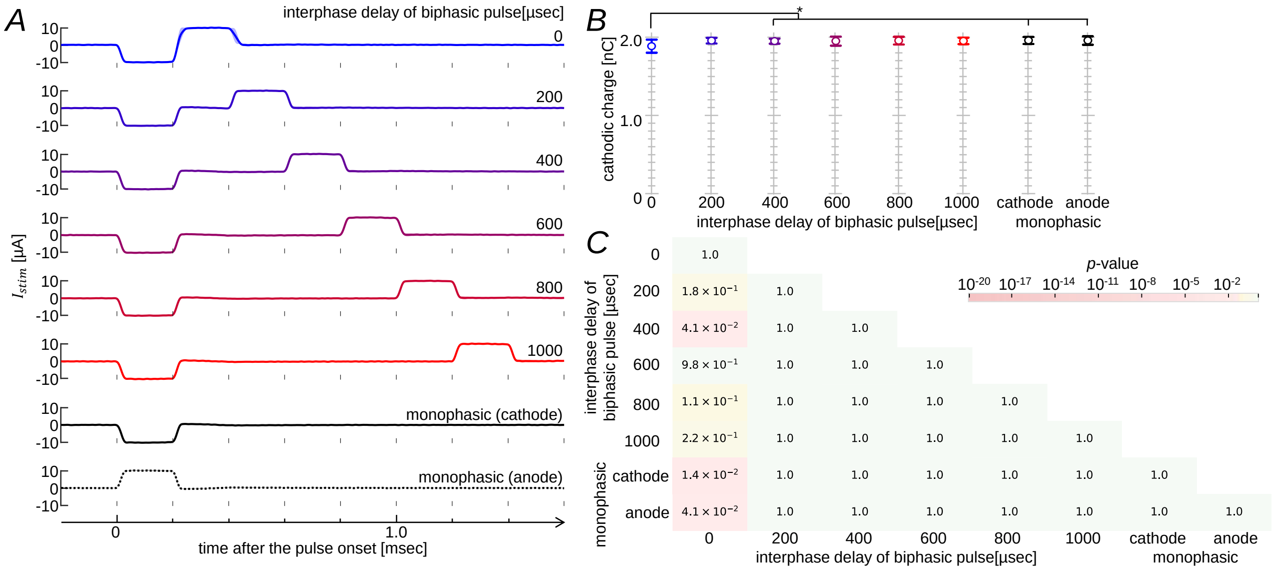

S-M-Figure 5. The current pulses used in the second and the third datasets. A) Traces of the stimulus current pulses. Lines represent the means, and shadings represent the s.d., across twelve tissue slices examined. B) Measured charge quantities in the first phases of the current pulses shown in A. Marks indicate the means, and error bars indicate the s.d., across the twelve slices. Statistics were first assessed with Friedman test, followed by Conover's post-hoc test with Holm correction. * indicates $p < 0.05$. C) Detailed statistical results for B. This matrix displays the p-values from Conover's post-hoc test (with Holm correction). The colormap indicates the level of statistical significance for each pairwise comparison; red-shaded cells indicate p < 0.05.

However, there was no significant difference in the stimulus charge among the other seven pulses (n = 12, Friedman test, $\chi^2(6) = 3.04$, $p = 0.80$). In the second dataset, the VSD signals were obtained under only the control condition. In the third dataset, the VSD signals were obtained under both control and synaptic blockade conditions.

In the fourth dataset, we applied a cathodic monophasic pulse train and cathodic-first biphasic pulse trains with different interphase delays to six tissue slices. The parameters of the cathodic monophasic pulse were set to 10 or 20 µA in current amplitude and 200 µsec in pulse duration. The parameters of the biphasic pulses were set to 10 or 20 µA/phase in current amplitude, and 200 µsec in phase duration. The interphase delay was set to either 0, 100, 200, 300, 400, 500, 600, 800, or 1000 µsec. The recorded traces of the current pulses are shown in S-M-Figure 6*A*-*B*. There was no significant difference in the stimulus charge among the ten pulses in each current amplitude (n = 6, Friedman test, 10-µA: $\chi^2(9) = 16.7$, $p = 0.054$, S-M-Figure 6*C*; 20-µA: $\chi^2(9) = 4.99$, $p = 0.84$, S-M-Figure 6*D*). The VSD signals were obtained under both control and synaptic blockade conditions.

11 Code availability

All original scripts used for the analyses in this paper are available at the following GitHub repository: https://github.com/Life-Assistive-Intelligence-Lab/2025_FrontiersInNeuroscience.git

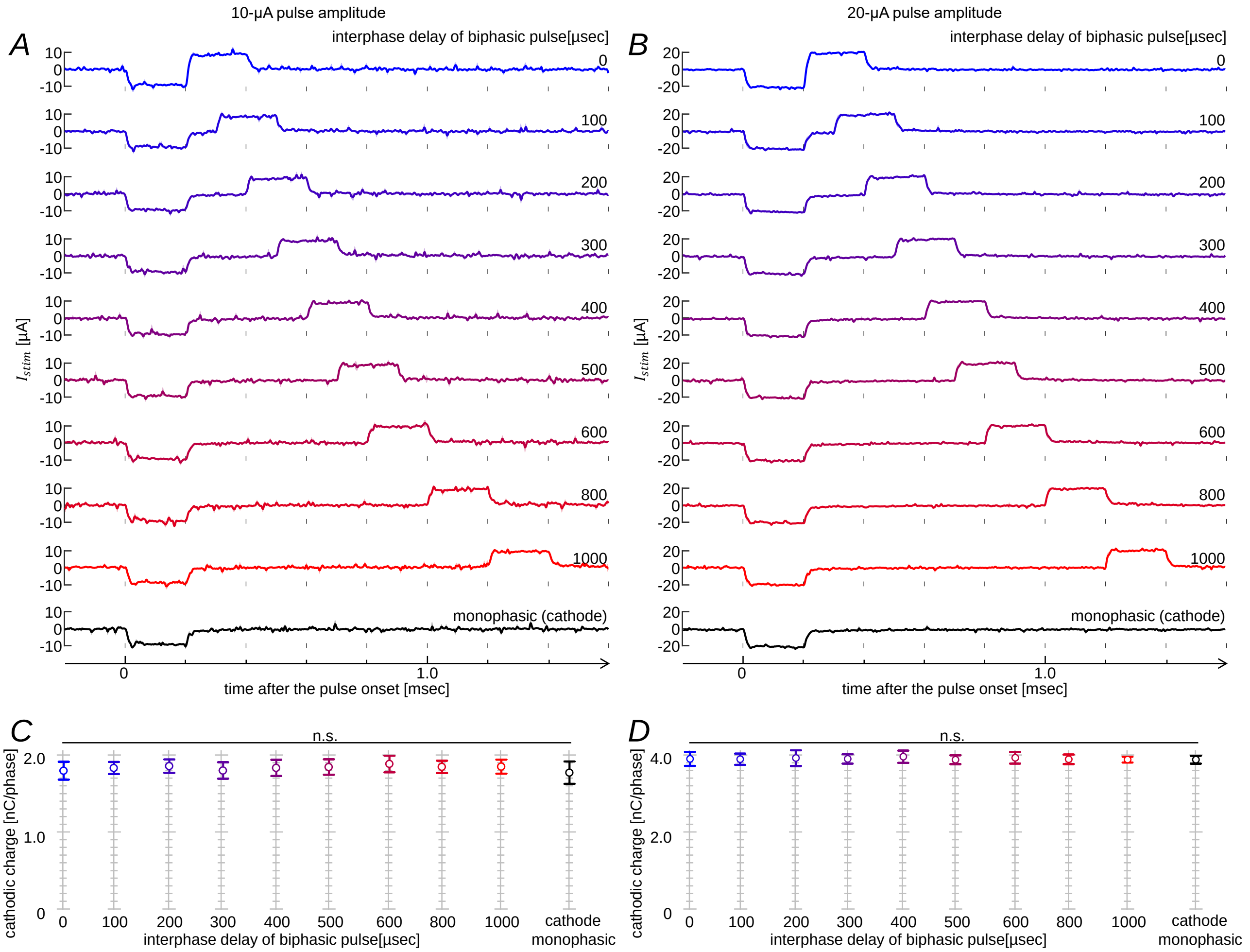

S-M-Figure 6. The current pulses used in the fourth dataset. A, B) Traces of the stimulus current pulses with 10-μA/phase (A) and 20-μA/phase (B) in current amplitude. Lines represent the means, and shadings represent the s.d., across six tissue slices examined. C, D) Measured charge quantities in the first phases of the current pulses with 10-μA/phase (C) and 20-μA/phase (D) in current amplitude. Marks indicate the means, and error bars indicate the s.d., across the six slices.
